# Chromosome-scale genome assembly and gene annotation of the hydrothermal vent annelid *Alvinella pompejana* yield insight into animal evolution in extreme environments

**DOI:** 10.1186/s12915-025-02369-7

**Published:** 2025-09-02

**Authors:** Sami El Hilali, Philippe Dru, Alan Le Moan, Yang I Li, Martijn A. Huynen, André Hoelz, Robert C. Robinson, José M. Martín-Durán, Didier Jollivet, Adam Claridge-Chang, Richard R. Copley

**Affiliations:** 1 Laboratoire de Biologie du Développement de Villefranche-sur-mer, Institut de la Mer de Villefranche-sur-mer, Sorbonne Université, CNRS UMR7009, Villefranche-Sur-Mer, 06230 France; 2 UMR 7144 AD2M, CNRS-Sorbonne Université, Station Biologique de Roscoff, Roscoff, France; 3 Section of Genetic Medicine, University of Chicago, Chicago, IL USA; 4 Department of Human Genetics, University of Chicago, Chicago, IL USA; 5 Department of Medical Biosciences, Radboudumc, Nijmegen, The Netherlands; 6 California Institute of Technology, Division of Chemistry and Chemical Engineering, 1200 East California Boulevard, 1200 East California Boulevard, Pasadena, CA 91125 USA; 7 School of Biomolecular Science and Engineering (BSE), Vidyasirimedhi Institute of Science and Technology (VISTEC), Rayong, Thailand; 8 Research Institute for Interdisciplinary Science, Okayama University, Okayama, Japan; 9 School of Biological and Behavioural Sciences, Queen Mary University of London, London, United Kingdom; 10 Program in Neuroscience and Behavioral Disorders, Duke-NUS Medical School, Singapore, Singapore; 11 Institute for Molecular and Cell Biology, A*STAR Agency for Science, Technology and Research, Singapore, Singapore; 12 Department of Physiology, National University of Singapore, Singapore, Singapore

**Keywords:** *Alvinella pompejana*, Deep-sea annelid, Hydrothermal vent, Vent populations, Metazoan thermotolerance, Physiological adaptation

## Abstract

**Background:**

The Pompeii worm *Alvinella pompejana*, a terebellid annelid, has long been an exemplar of a metazoan that lives in an extreme environment, on the chimney wall of deep-sea hydrothermal vents, but this very environment has made it difficult to study. Comprehensive assessment of *Alvinella pompejana* genome content, and the factors that could explain its ability to thrive in seemingly hostile conditions has been lacking.

**Results:**

We report the chromosome-level genome sequence of *Alvinella pompejana* and population-level sequence variants. We produced a set of gene models and analysed the predicted protein set in the light of past hypotheses about the thermotolerance of *Alvinella*, comparing it to other recently sequenced vent annelids. Despite its extreme environment, we find evidence for relatively conservative evolution of protein amino acid composition and genome evolution as measured by synteny. We suggest that prior hypotheses of loss of amino acid biosynthesis genes associated with obligate symbioses reported in siboglinid annelids are mistaken, and that *Alvinella* and siboglinids are typical metazoans in this regard. *Alvinella* encodes a number of respiratory enzymes unusual for bilaterian animals, suggesting an ability to better tolerate hypoxic environments. We find evidence of a parallel increase in the number of globin encoding genes and loss of light sensitive opsins and cryptochromes in deep-sea annelids.

**Conclusions:**

Our results provide a comprehensive *Alvinella* protein and genome resource and shed light on the adaptation of *Alvinella* to temperature, hypoxia and darkness, as well as cryptic speciation, giving a firm base from which future studies can be taken forward.

**Supplementary Information:**

The online version contains supplementary material available at 10.1186/s12915-025-02369-7.

## Background

The Pompeii worm, *Alvinella pompejana* Desbruyères & Laubier, 1980, inhabits the hottest part of the hydrothermal vent environment along the East Pacific Rise. At depths of several kilometres, these vents release superheated, metal-rich water, potentially exposing *Alvinella* to toxic chemicals and extreme temperature and pressure. The thermal optimum for *A. pompejana* is likely to be beyond 42 °C but below 50 °C [1], and the related alvinellid *Paralvinella sulfincola* has been shown to prefer temperatures in the 40–50 °C range and tolerate 55 °C for brief periods [2]. These compare with body temperatures in excess of 45 °C that have been reported in some species of birds [3, 4], with the further caveat that there is likely to be an interplay between behaviour and the fluctuating temperatures of vent environments [5]: *Alvinella* is thought to moderate local water temperature via movement in and out of its tube [6].

*Alvinella pompejana* has held enduring interest as a source of stable metazoan proteins suitable for structural and functional studies [7–13], but in part owing to their inaccessibility, molecular work on alvinellids has been limited. Relatively large transcript sets have been constructed [8, 14] and attempts to correlate protein amino acid composition with thermotolerance have been a recurring theme, e.g., [8, 15]. Most recently, Jollivet and co-workers analyzed orthologous loci from 6 alvinellid species, inferring that the ancestral alvinellid was likely thermophilic and that cold-water adapted alvinellids showed distinct amino acid preferences [16].

The alvinellids, with 14 species currently described, are members of the terebellid clade of annelids [17, 18]. Another kind of annelid, the vestimentiferan tubeworms from the siboglinid family, are also found in deep-sea hydrothermal vents and cold seeps, but live in colder habitats. Recently, the genomes of three of these siboglinids have been reported [19–21], along with a fourth non-vestimentiferan, the idiosyncratic bone-eating *Osedax frankpressi* [22]. Siboglinids are unusual among animals in lacking a digestive system. Instead, they have a trophosome, housing symbiotic bacteria that oxidize hydrogen sulfide to generate ATP and supply nutrients to the host. Alvinellids, in contrast, are grazers with an apparently intact digestive system [23] and epibiotic symbionts [24]. Terebellid and siboglinid annelids are not closely related [25], suggesting that their colonisation of deep-sea vents has occurred independently and raising the question of how their strategies to adapt to the deep-sea vent environment differ.

Recently, a fragmented whole genome sequence of *Alvinella pompejana* was analysed together with its transcriptome to explore gene diversification patterns of *A. pompejana* and the closely related *A. caudata* [26]. A sub-chromosome-level genome of *Paralvinella palmiformis* has been reported and analysed within the context of an epigenome study, without extensive study of its protein coding gene set [27]. To enquire further into the nature of its thermotolerance and deep-sea adaptations, here we report a chromosome-level genome assembly of *Alvinella pompejana*. We study the genome-wide population structure of resequenced individuals collected from two distinct genetic units on each side of the Equator [28, 29] and note the existence of semi-permeable barriers to gene exchanges between these two units. We perform analyses of phylogeny, genomic synteny and protein amino acid composition, situating *Alvinella* within the context of other annelids, and then perform more detailed analyses of specific protein families related to thermal vent adaptation and physiological tolerance of vent conditions. We pay particular attention to similarities and differences with vent-dwelling siboglinid annelids and other relevant taxa.

## Results

### Overview of the nuclear genome

Following extraction of high molecular weight genomic DNA, we sequenced and assembled a high-quality genome from a single individual (see the “Methods” section). Further scaffolding of the assembly using sequence from Hi-C and Chicago libraries (Dovetail Genomics, using further animals, see methods) resulted in a 367.4 Mb genome (N50 = 21.45 Mb) (Additional File 1: Table S1) out of which 95% (348.5 Mb) is covered by 17 pseudo-chromosomes (with scaffolds longer than 1 Mb) (Additional File 1: Table S2). The size of the assembly is in line with previously reported measures using flow cytometry [30]. Dixon and co-workers report a karyotype of 16 chromosomes for *Alvinella pompejana*, although they note sampling difficulties and poor quality of chromosomes [31]. Our 17 chromosomes appear well supported on the Hi-C contact map (Additional File 2: Fig. S1), but analysis of synteny data raises the possibility of an artefactual break between our pseudo-chromosomes 10 and 17 (see later).

**Fig. 1:**
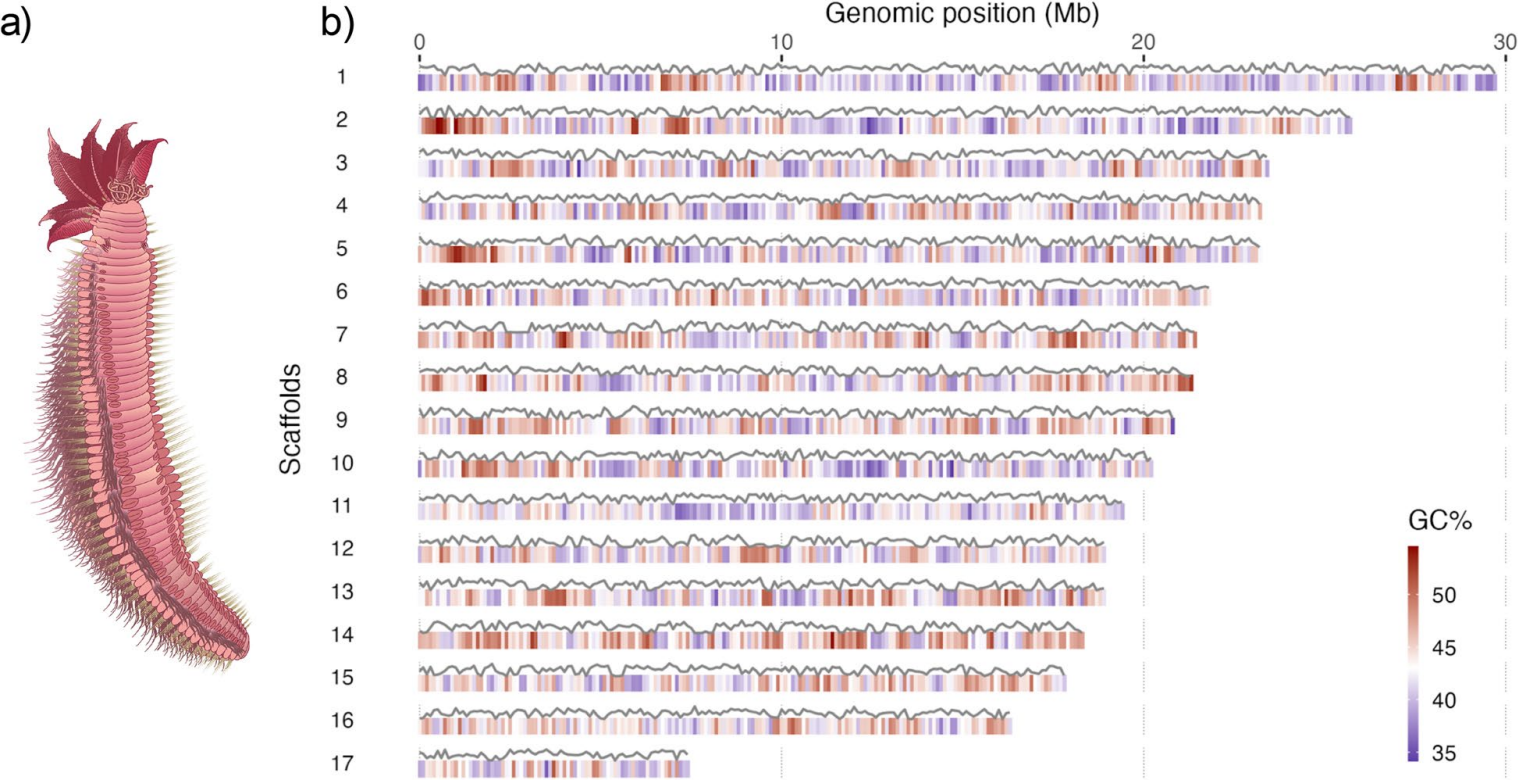
*Alvinella* and its chromosomes. **a** Schematic of *Alvinella pompejana* (~ 10 cm). **b** Pseudo-chromosomes of *Alvinella* genome assembly. The GC content (red/blue colour ramp) and gene density (line) over 100 kb windows are shown, with the latter computed as the percentage of the coverage by predicted genes in 100 kb non-overlapping windows, with the top line indicating 100%

The genome shows a GC content of 43.3%, the highest level yet seen in annelids (Additional File 1: Table S1). Its repetitive content is 23.3%, lower than other sequenced annelid genomes, with the exception of the ‘miniaturized’ genome of *Dimorphilus gyrociliatus* [32] (Additional File 1: Table S1). Our assembly was 94.4% (BUSCO5 metazoa odb10) complete compared to the currently available metazoan gene set, in line with recently released high-quality genomes of annelids. We used RNA-seq data to support gene predictions and produced a set of 19,179 protein-coding gene models. This gene set was 96.5% complete (BUSCO5 metazoa odb10) (Fig. 1).

### The mitochondrial genome

**Fig. 2:**
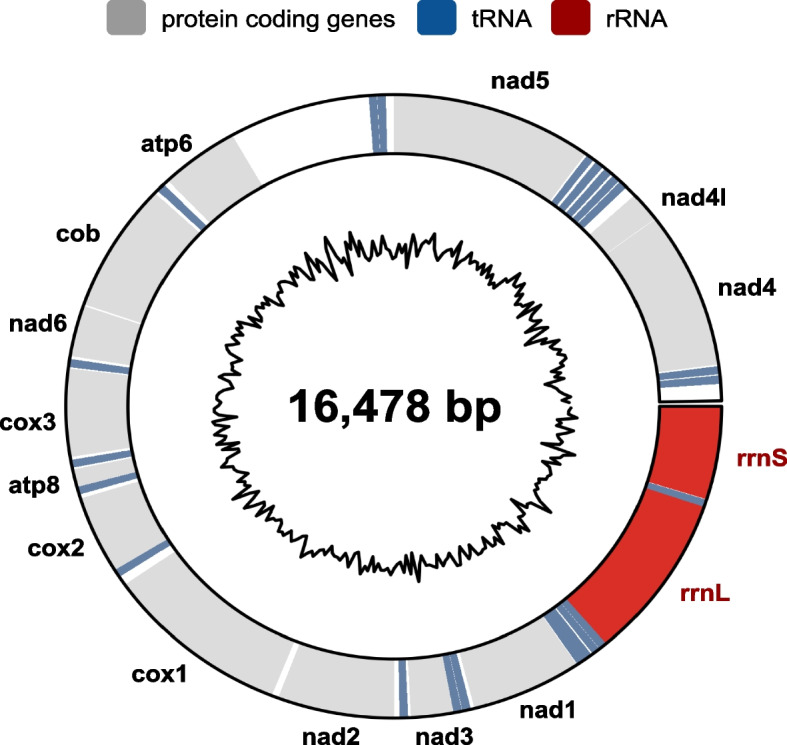
Fully assembled mitochondrial genome. The inner circle indicates the GC content calculated over adjacent genomic windows of 50 bp. Drawing using the circlize R package [37]

*Alvinella pompejana* tolerates a broad range of temperature [33]. A study of marine invertebrates showed that mitochondria of deep-sea species living in zones with a high and highly variable temperature were more resistant to high temperatures than those of other species living in cooler deep-sea and shallow water environments [34]. Mitochondrial respiration of *Alvinella* was reported to be the most stable when compared to other hot-adapted species, including the siboglinid *Riftia pachyptila* and the brachyuran crab *Bythograea thermydron*. We find the full mitochondrial genome of *Alvinella* consists of a 16.478 kb circular contig showing a GC content of 41%. Its relative order of protein coding and ribosomal RNA genes is identical to the recently released mitochondrial genomes of the siboglinid *Riftia pachyptila* [21], and the alvinellid *Paralvinella palmiformis* [35] and the highly conserved order in Pleistoannelida [36] (Fig. 2).

### Geographical differentiation of the genome

**Fig. 3:**
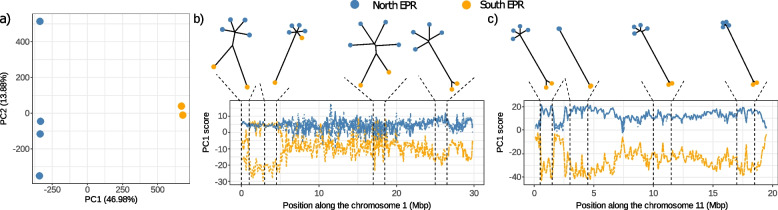
Genome-wide population structure of *A. pompejana* explored across two EPR South samples (in orange) and four EPR North samples (blue). The graph in **a** shows a principal component analysis (PCA) performed on the six samples genotyped at 3.33 M SNPs. Most of the variation is captured by PC1 (PC1 = 46.98%; PC2 = 13.88%) and distinguishes samples from either side of the equator. Graphs in **b** and **c** show detailed analyses of genomic regions with contrasted patterns of divergence in **b** chromosome 1 and **c** chromosome 11. In each subplot, the top graphs show phylogenetic trees computed from the polymorphism data extracted from 1Mbp to 1.5Mbp windows defined from the patterns of divergence observed in the local PCA plot at the bottom. In the local PCA plots, each line follows the individual PC score of each sample over a sliding window. The vertical dotted lines show the limits of the genomic regions that were used to infer the phylogenetic trees. On chromosome 1, the phylogenies showed, in order of appearance, a region of introgression where one EPR South sample is located at mid-distance from the EPR North samples and the other EPR South sample, a region of introgression where one EPR South sample clustered perfectly with the EPR North samples, a region of weak divergence where all samples were similarly distant, and a region of high divergence where individuals on either side of the equator were clearly separated. On chromosome 11, all phylogenetic trees showed clear North–South EPR separation, with the second and last trees also showing small terminal branches expected from low genetic diversity within each population

Genetic studies of Pompeii worm populations along the East Pacific Rise (EPR) have shown two distinct genetic units on either side of the Equator [28, 29], with the existence of a hybrid zone around 7°S/EPR. We confirmed this finding here using 3.33 million SNPs obtained after the WGS data processing. The genetic differentiation between individuals on either side of the Equator was much higher than expectation based on the divergence of genes [26] (Fig. 3a), with an average 70% polymorphism identity between individuals of the same population but 50% between the southern and northern populations. The differentiation is, however, unevenly distributed with some portions of chromosome highly differentiated and others not (Fig. 3b). Both individuals sampled in the south EPR are introgressed by alleles from the north, demonstrating that hybridization of the two geographic forms extends south of the hybrid zone described around 7°S/EPR. The introgression footprints are often located in different genomic regions across the two introgressed individuals (Additional File 2: Fig. S2). In addition, the introgressing alleles are distributed in blocks along the genome (Fig. 3b). This pattern suggests the presence of non-recombining regions where long and divergent haplotypes are maintained for significant periods after their introgression. Chromosome 11 shows a remarkably contrasting pattern of divergence between the north and the south EPR (Fig. 3c), with homogeneous divergence found over the entire chromosome, no signs of introgression and lower genetic diversity (H_obs_ C11 = 0.19 vs H_obs_ overall = 0.38). We suggest that this chromosome acts as a major genetic barrier between the North and South EPR populations of *A. pompejana*.

### Evidence of a symbiont

Given the complexity of the vent ecosystem and the prevalence of symbioses, we were mildly surprised to find that only 574 out of the original 19,179 predicted protein sequences had a bacterial best hit when searched against the NR database of the NCBI (see methods). By far the majority of these (455 out of the 574) were encoded on sequences that did not form part of the 17 pseudo-chromosomes, although these non-pseudo-chromosomal sequences encode far fewer proteins (919 vs 18,260 on the pseudo-chromosomes), so we suspect they represent bacterial contigs rather than genomically integrated horizontal gene transfer events. We sequenced a tissue sample taken from adult gills (see methods). As episymbionts are found mainly on the dorsal surface of the worm body, it seems likely that we have avoided bacterial sequencing to a large extent. In contrast, an earlier draft of the *A. pompejana* genome assembled from different sequence reads does appear to contain an extensive set of proteins from Bacteria (see methods and Additional File 1: Table S6), with best hits including sequences labelled as *Alvinella pompejana* symbionts, epsilonproteobacteria (Campylobacterota), the sulfate reducing *Desulfobulbus* and metabolically significant genes such as dissimilatory sulfite reductase (Additional File 1: Table S7) [26, 38, 39].

Halanych and co-workers presented an analysis of the presence and absence of amino acid biosynthetic genes in the siboglinid *Lamellibrachia luymesi* and concluded that it lacks many genes essential for amino acid biosynthesis, contrasting it with *Capitella teleta*, which they claimed encoded many of these missing genes [19]. This finding is repeated in the report of the genome sequence of *Riftia pachyptila* [21] and in both cases regarded as consistent with loss of the enzymes and a dependence of siboglinids on their bacterial symbionts for amino acids and co-factors. The absence of these amino acid biosynthetic pathways is, however, a synapomorphy of the Metazoa [40–43]; so, if true, their reported presence in *Capitella* and hence inferred loss in the lineage leading to siboglinids would be a remarkably unparsimonious environmental adaptation.

We therefore reanalysed those putative *Capitella* protein sequences, without *Lamellibrachia* counterparts, that form the basis for the hypothesis. We found that most are very similar to bacterial proteins (Additional File 1: Table S4), typically an *Endozoicomonas* species [44], but none have credible annelid, or in most cases, even animal orthologs (except in two cases where they appear to have been overlooked by Li et al.). Although it is possible that a proportion of these represent horizontal gene transfer into the *Capitella* genome (or an unknown symbiont), it seems likely that they are bacterial contaminants in the *Capitella* data [45]. If this interpretation is correct, it suggests that the repertoire of amino acid biosynthesis pathways in siboglinids is not exceptional among annelids or animals.

### Phylogenetic placement and synteny in the context of annelids

**Fig. 4:**
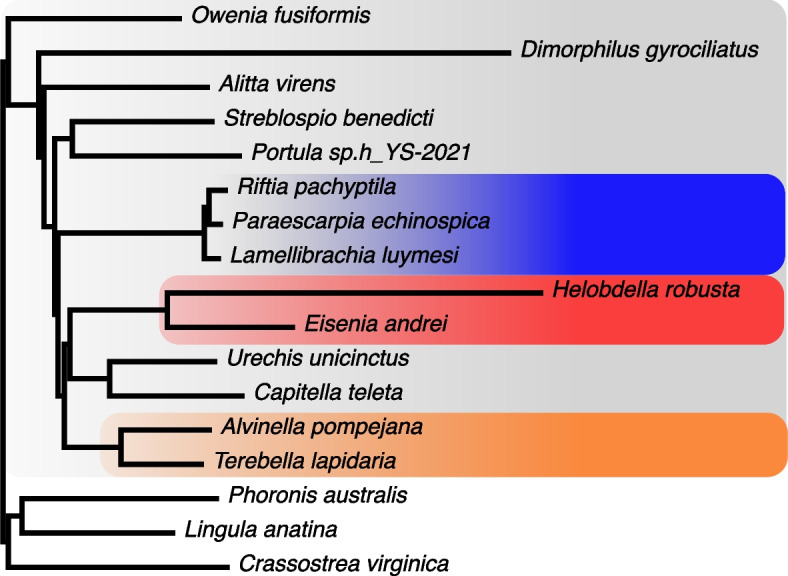
Phylobayes CAT + GTR phylogenetic tree of representative annelids with complete genomes. Calculated from a concatenated alignment of protein sequences corresponding to 339 orthologous genes from 14 annelid taxa and 3 outgroups (118,092 positions, see methods). The annelid clade has a grey background, and within that, siboglinids blue, clitellates red and terebellids orange. *Lingula anatina* (brachiopod) and *Crassostrea gigas* (mollusc) are shown as outgroups. All nodes have a posterior probability of 1

We used phylobayes to calculate an annelid phylogeny using the CAT + GTR model of sequence evolution (Fig. 4) [46], focusing mainly on Sedentaria, of which *Alvinella* is a member [47]. The tree demonstrates the distinction between *Alvinella pompejana* and the siboglinids (*L. luymesi*,* P. echinospica*,* R. pachyptila*). Our analysis groups *Alvinella* with *Terebella lapidaria* as the terebellids, which are sister to a clade of the clitellate annelids (*H. robusta* and *E. andrei*) and Capitellidae/Echiuridae, as in [47]. The branch lengths separating these clades are extremely short, though, and have not been resolved consistently with different molecular datasets [47–49]. It is notable that neither the *Alvinella* nor siboglinid branch lengths show the rapid evolution of the clitellates or *Dimorphilus*, and further, a deep-sea ancestor for the combined clade including siboglinids and terebellids is unlikely on parsimony grounds.

**Fig. 5:**
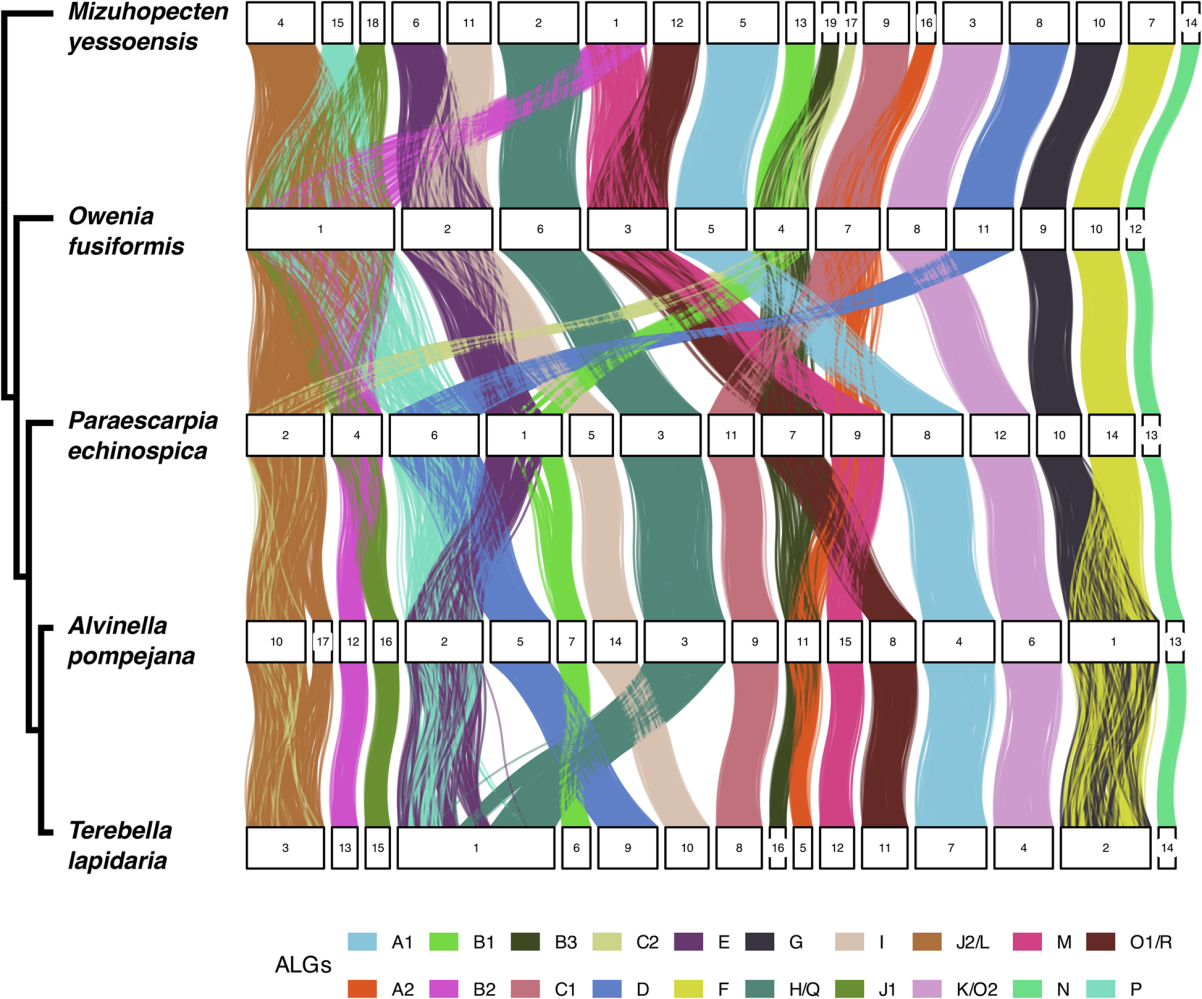
Chord diagram of syntenic relationships between annelid chromosomes. The terebellid annelids *Alvinella *and* Terebella* are compared to the siboglinid vent worm *Paraescarpia*, the deep branching annelid *Owenia fusiformis* and the mollusc *Mizuhopecten yessoensis*. Colour-coding follows ancestral linkage groups assignments (ALGs) (see the “Methods” section)

We used our chromosome-scale assembly to examine conserved synteny between *Alvinella*, other annelids and a mollusc outgroup by equivalencing orthologous genes [50]. Our analysis showed well-conserved synteny between *Alvinella* and *Branchiostoma* (Additional File 2: Fig. S3), indicative of good conservation of chromosomes between *Alvinella* and the protostome / deuterostome ancestor. Synteny conservation with other annelids and inferred ancestral linkage groups (ALGs) is shown in Fig. 5 [49]. Our assembly contained 17 segments longer than 1 Mb. An earlier determination of karyotype reported 2n = 32 chromosomes [31]—this would be consistent with an artefactual split between our chromosomes 10 and 17, which both correspond to the J2/L ALG. *Alvinella* shares fusions diagnostic for annelids, and also a fusion of ALGs F and G with *Terebella lapidaria*, which is thus likely a synapomorphy for terebellid annelids. *Alvinella* does not show any of the siboglinid-specific fusions discussed in [49]. The overall picture of relatively conservative synteny evolution is similar to other marine annelids, but in marked contrast to e.g. *Dinomorphilus* or clitellates [32, 49].

### Bulk amino acid composition of *Alvinella* proteins

**Fig. 6:**
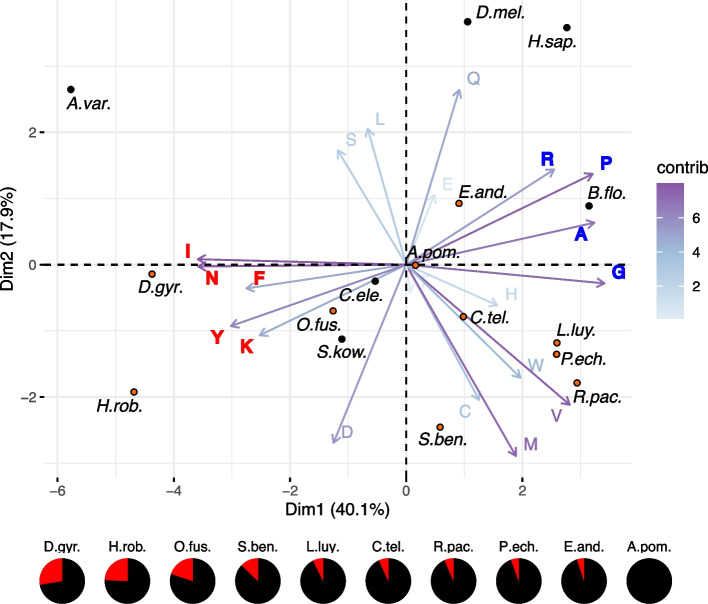
PCA of amino acid composition of various annelids and other Metazoa. Annelid species are indicated by orange dots. Arrows show loading vectors for amino acids (i.e. the extent to which they contribute to the illustrated dimensions)—the darkness (closer to purple) indicates the extent of their contribution. Dimension 1 is dominated by the GC content of codons encoding the amino acids, with low GC (YFINK—red) and high GC (GARP—blue) amino acids at opposite extremes. Genomic GC content as a percentage of the GC content of *Alvinella* (black) for each annelid is shown below the plot, with extra AT content (red) making up the remainder (see Additional File 1: Table S1). Non annelid taxa: A.var, *Adineta vag*a; B.flo, *Branchiostoma floridae*; C.ele, *Caenorhabditis elegans*; D.mel, *Drosophila melanogaster*; H.sap, Human; S.kow, *Saccoglossus kowalevskii*

The thermophily trait of the Pompeii worm has previously been proposed to rely on the amino acid composition of its proteins, with preferential replacements similar to those observed between thermophilic and mesophilic bacteria [8, 14–16]. We compared the amino acid composition of the predicted proteins of orthologous genes from a variety of Metazoa, including ten annelids of which four were vent species (see the “Methods” section). Principal component analysis of these compositions showed a dominant axis correlating with GC content of the codons encoding amino acids and roughly following the trend of genomic GC content (Fig. 6). Far from being an outlier though, *Alvinella pompejana* has the closest Euclidean distance to the intersection of the two axes. Despite the fact that it has the highest GC content (43%) of the sequenced annelids, its position contrasts strongly with the other vent annelids of lower GC content (~ 40%). That amino acid usage tracks GC content has long been noted [51]. In an analysis of bacterial genomes, Kreil and Ouzounis observed a similar trend, but their second principal component neatly distinguished thermophilic from non-thermophilic species [52], whereas we do not see an obvious trend on our 2nd axis.

### ANTP-like homeoboxes and other transcription factors

**Fig. 7:**
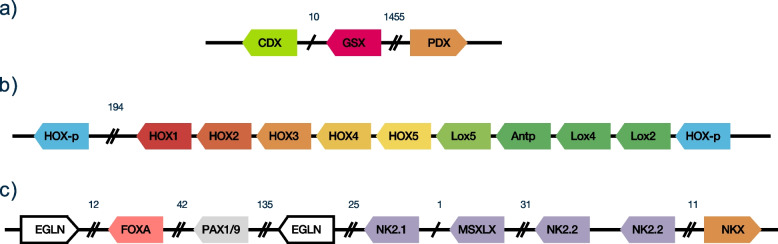
*Alvinella* Parahox, HOX and pharyngeal gene clusters. **a** Parahox cluster (APscaff0001). **b** HOX cluster; several likely spurious transcripts are not shown in the posterior part of the main cluster (APscaff0012). **c** Genes of the deuterostome pharyngeal cluster (APscaff0004). MSXLX was not identified in the gene build, but is present in the genome sequence. Numbers above intergenic regions are approximate numbers of intercalated genes

*Alvinella* contains an annelid-like homeobox cluster of around 1 Mb in length. In contrast to siboglinids [22], an ANTP gene is present (Fig. 7). These genes are on the same scaffold as DLX as well as MNX, GBX and EN (the components of the so-called EHGbox), as in *Platynereis dumerilii* [53]. Unlike siboglinid annelids [21], only one copy of Engrailed is present. The parahox genes CDX and GSX are within 100 kb of each other, with PDX more distant, showing some disruption of the likely plesiomorphic state. Interestingly, the transcription factors of the so-called pharyngeal cluster, a proposed deuterostome synapomorphy [54], including PAX1/9, FOXA, MSXLX and NKX2.1/2.2 are all present within around 250 genes of each other on the same scaffold, adding support to the possibility that this organisation is in fact plesiomorphic for the Bilateria [55]. A variety of NK-like homeodomain proteins are encoded on a further scaffold (APscaff0014: EMX, NK2-3/5/6, NK3, MSX, VAX, LBX, TLX, NK1, HMX, HHEX, NOTO, NK6 and NK7) with varying degrees of proximity, suggestive of breakup of a tighter previous degree of clustering, as proposed for the NK-cluster of the ancestral bilaterian [56].

Among Forkhead transcription factors, we noted the presence of FOXI. Although found in *Capitella* and some other annelids, it is not present in siboglinid genomes [57] and is typically absent in protostomes. FOXI is associated with gill structures in deuterostomes and is a marker for ionocytes [58–60]. Both siboglinids and alvinellids have extensive gill structures involved in gas transfer, but the shared presence of FOXI in *Alvinella* and deuterostomes hints at the presence of shared ionocyte cell-types lost in other taxa.

### Protective structures: cuticle and tube composition

The cuticle of *Alvinella* has been noted to have unusual supercoiled collagen fibrils [61]. Animal genomes encode many collagens, structural proteins composed of multiple repeating triplets of Gly-Xaa-Yaa amino acids. Three collagen chains combine to form a triple helical structure. Thermal stability has been related to the presence of 4-hydroxyproline at the Y position of the repeat [62]. An *Alvinella* interstitial collagen has been highlighted as a protein of unusual thermal stability [63]. We identified this protein (Genbank accession AAC35289.2; 890 residues) as a truncated version of one of our predictions (APscaff0012.263.p1; 1483 residues). We identified 42 predicted *Alvinella* proteins containing hits to the Pfam Collagen HMM (see methods) several of which are extremely long, with the longest having 8859 amino acids. Although it is difficult to rule out that the repetitive nature of collagen molecules might have led to genomic misassembly artefacts, we detected similarly long proteins in assembled transcriptomes of *P. pandorae* (8689 aa), *P. grasslei* (8413 aa) and *P. fijiensis* (7719 aa) (Table 1). Notably, no collagens from the recently sequenced siboglinid gene predictions reach a similar length. The earthworm *Eisenia andrei* does include such a collagen, but, as has been noted before in other earthworms, has a much higher proportion of proline at the Xaa position, which is not associated with thermal stability [62, 64]. Although we cannot quantify 4-hydroxyproline content from protein sequence alone, the high proline counts at the Yaa position for *Alvinella* and its overall high proline content are consistent with increased thermostability. These trends in collagen proline content run counter to the global amino acid usage that might be expected from the PCA plot presented earlier.

**Table 1:** Annelid collagen-like proteins with the most GXY repeats. Repeats were counted for each match to the pattern shown at the head of the second column. The proportion of repeats with a proline at positions X and Y are shown. P_gra, A_pom, P_pan and P_fij are alvinellids; R_pac, L_luy and P_ech are siboglinids; E_and is an earthworm. P_pan and P_gra are cold adapted annelids, whereas P_fij and A_pom live on vent chimney walls [16]

	Gxx**GXY**GxxG	**G-P-Y %**	**G-X-P %**
P_gra|DN108_c0_g3_i3-orf	5722	0.386	0.264
**A_pom|APscaff0012.353.p1**	**5698**	**0.372**	**0.333**
E_and|GWHPACBE014886	5660	0.403	0.215
P_pan|DN152_c0_g2_i2-orf	5628	0.331	0.217
P_fij|DN15_c0_g4_i1-orf	5012	0.332	0.326
R_pac|jg13387.t2	840	0.133	0.362
L_luy|FUN_004270-T1	800	0.09	0.34
P_ech|Scaf3834_5.3	684	0.211	0.231

**Fig. 8:**
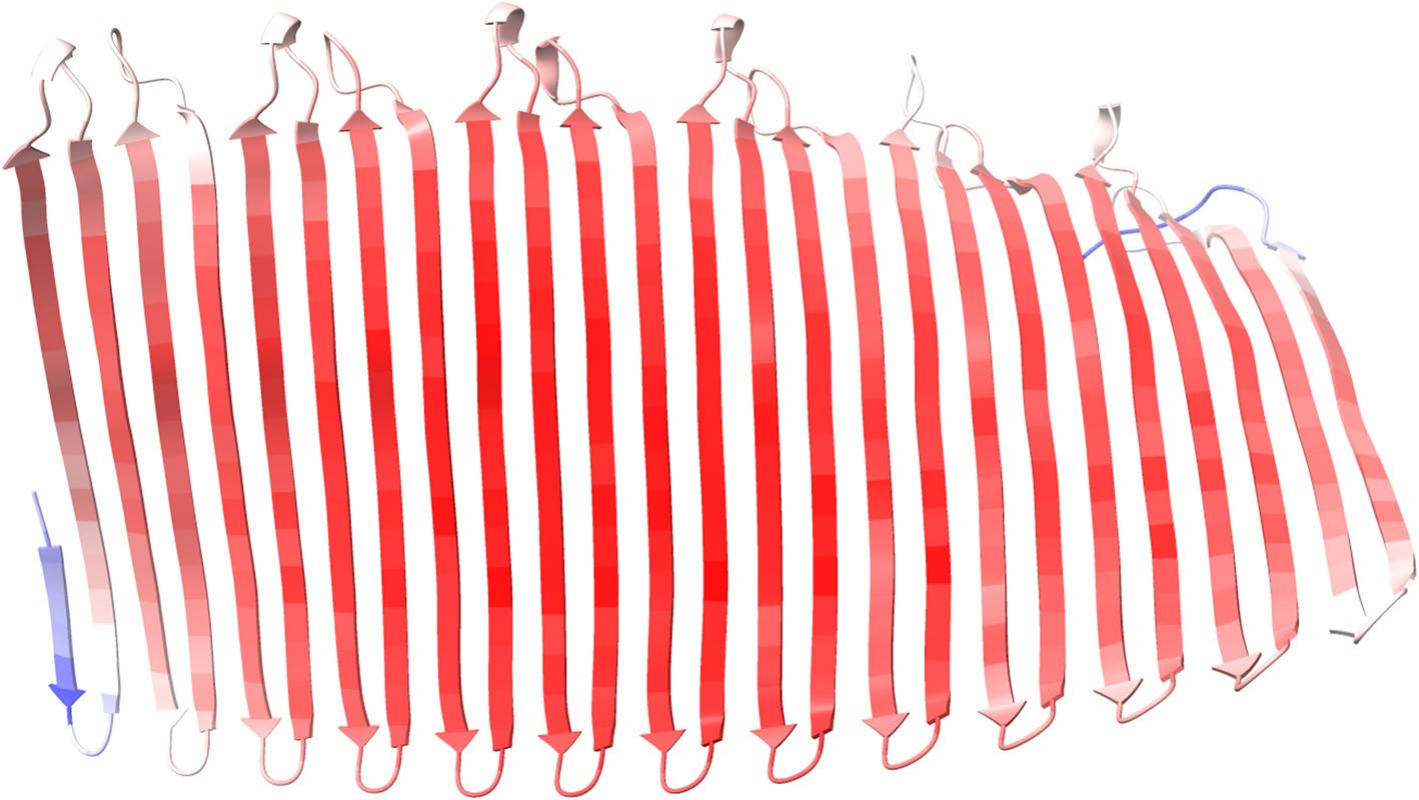
Extended β-sheet structure of the *Alvinella* protein with S,G,A amino acid composition most similar to the reported values for tube composition. The strands are 17 amino acids long. Predicted using Alphafold2, coloured by pLDDT values, red = high, i.e. more confident

*Alvinella* builds a stable, watertight, parchment-like tube, enabling it to regulate its thermal microenvironment by drawing in cooler water with up and down body movements. Gaill and Hunt investigated the amino acid composition of these tubes, finding them made of a glycoprotein matrix, in contrast to the chitin/proteoglycan mix of the siboglinid *Riftia* [65], and Vovelle and Gaille reported secretions rich in sulphated glycosaminoglycans [66]. Amino acid composition revealed an abundance of the small amino acids serine, glycine and alanine, in a manner reminiscent of silk fibroins [65, 67] and barnacle shell proteins [68]. For all *Alvinella* proteins, we calculated the Euclidean distance to the tube percentages of serine (25%), glycine (20.6%) and alanine (11.8%). The best scoring match was confidently predicted to form an unusually large extended β-sheet structure (Fig. 8), and although speculative, it is possible that this and similar proteins oligomerize as a fibrillar structural component of the tubes.

In other annelids, the Tyrosinase gene family has been implicated in the modification of tyrosine-rich protein families to enable DOPA-mediated cross-linking of ‘glue’ anchor proteins, and tyrosinase gene family expansions have been observed [69]. *Alvinella*, however, encodes no matches to the Pfam Tyrosinase domain (see the “Methods” section).

### Globins and oxygen transport

In common with other annelids [70, 71], the *Alvinella* genome encodes a high number of globin genes, predicted to include both intra- and extra-cellular proteins. We detected 2 genomic clusters of globins corresponding to *Alvinella pompejana* specific gene duplications. The first, of 3 members (APscaff0001.1370.p1, APscaff0001.1371.p1, APscaff0001.1372.p1) is predicted to encode extracellular globins, each with the key histidine residues that coordinate the Zn^2+^ ions proposed to bind H_2_S [72]. The second genomic cluster, of 5 adjacent globins, encoded proteins lacking signal peptides, which are therefore unlikely to be extracellular. Among our annelid data, they were most closely related to *Eisenia* proteins, with the tardigrade kumaglobin being the closest known structure [73].

Within the large clade of annelid extracellular globin genes, we identified further *Alvinella-*specific gene duplications, for a total of 11 genes. These *Alvinella-*specific duplications are independent of the main expansion of ‘B1’ type globins found in siboglinids [19–21]. A single *Alvinella* gene had a free cysteine similar to ‘B2’ type annelid globins (C + 1), while the genomic cluster of 3 members all had an ‘A2’-like free cysteine (C + 11), compatible with proposed H_2_S transport (Additional File 2: Fig. S4) [74]. *Alvinella* further encodes 4 linker genes involved in scaffolding the giant extracellular globin complex [75], a number comparable to other annelids, although these again appear to be related by independent duplication and loss events rather than straightforward orthology.

Another unusual globin with dehaloperoxidase activity has been characterised from the terebellid annelid *Amphitrite ornata* [76] and the similarity of an *Alvinella* globin noted [77]*.* We identified 4 unclustered but paralogous *A. pompejana* genes with a likely orthologous relationship to this gene, but no equivalent siboglinid sequences. In *A. ornata*, it has been speculated that these enzymes detoxify bromoaromatic compounds released by species living in close proximity (*Notomastus lobatus,* a polychaete and *Saccoglossus kowalevskii*, a hemichordate) [78]. In alvinellids, they may have a similar role in detoxifying vent chemicals or bacterial metabolites.

The unrelated oxygen transport protein, hemerythrin, was not detected in the *A. pompejana* proteome, or alvinellid proteins, despite its frequent occurrence in annelids, including siboglinids, and a previous report in *Paralvinella palmiformis* (Genbank accession KY007423.1); [79]. Further analysis of the *P. palmiformis* sequence showed it to be identical at the protein level to a sequence from the unrelated annelid *Oenone fulgida*, suggesting the possibility of sample contamination and the genuine absence of this family from alvinellids. The hemerythrin family is expanded in *Paraescarpia* and siboglinids generally compared to other marine annelids, and it is possible that it has there acquired a more significant role in oxygen transport [20].

### Hypoxia and anaerobic respiratory pathways

The increased numbers of globins observed in annelids, relative to other Metazoa, are suggestive of demanding oxygen availability. Some taxa undergoing periodic hypoxia (tardigrades, barnacles and copepods) have lost components of the HIF hypoxic response pathway [80, 81]. In *Alvinella* and siboglinids, we detected a full complement of HIF pathway genes (HIF1, HIF1AN, VHL and EGLN). HIF orthologs included the conserved motifs embedding the key proline that is hydroxylated and then bound by VHL prior to destruction [82] (Additional File 2: Fig. S5).

**Fig. 9:**
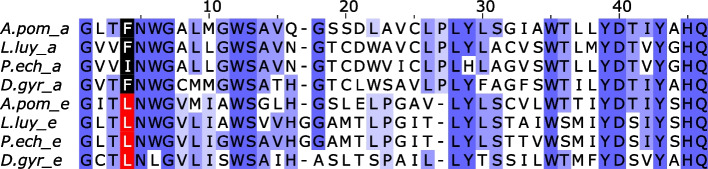
Tandemly duplicated exons in a selection of annelid COQ2 genes. ‘e’-type exons are implicated in rhodoquinone synthesis and are present in *Alvinella* and siboglinids. Tan and co-workers [87] highlight two residues differentiating these exons—our data suggest that the first of these is most diagnostic (red/black colouring)—the second (third residue from C-terminus, A/S in Tan et al.) does not show a clear pattern with our species sampling. Purple shading reflects the degree of sequence identity for the column. A.pom = *Alvinella pompejana*; L.luy = *Lamellibrachia luymesi*; P.ech = *Paraescarpia echinospica*; D.gyr = *Dimorphilus gyrociliatus*

We did, however, uncover other genomic evidence for tolerance of limited oxygen environments, finding that the *Alvinella* genome encodes a pyruvate:NADP + oxidoreductase (PNO), a marker of eukaryotic anaerobic pyruvate metabolism [83] and a soluble fumarate reductase (similar to OSM1 and FRD1 in *S. cerevisiae*) (supplementary information). These genes were also present in siboglinids and other annelids, as well as some non-chordate bilaterian taxa. In yeast, OSM1 and FRD1 have been implicated in maintaining an oxidising environment under anaerobic conditions [84]. In *Alvinella*, we also detected genes for two octopine dehydrogenase-like proteins (supplementary information). These catalyse opine synthesis, the reductive condensation of pyruvate with arginine (yielding octopine) alanine (alanopine) or glycine (strombine), allowing the anaerobic generation of ATP. This pathway may be advantageous relative to lactate formation as it is osmotically neutral [85]. Octopine dehydrogenases have a sparse phylogenetic distribution, found in cnidarians, molluscs and some annelids. Notably, we could not detect them in siboglinids. Use of rhodoquinone as an electron donor for fumarate is another diagnostic of anaerobic metabolism [85, 86]. In animals, its synthesis requires a co-enzyme Q2 (COQ2) isoform including the ‘e-form’ exon, which is spliced in a mutually exclusive manner with an ‘a-form’ exon [87]. We found both the ‘a-form’ and ‘e-form’ exons of COQ2 adjacent within the *Alvinella* genome (Fig. 9), and transcript evidence from other alvinellids that mutually exclusive splicing occurs. Finally, we detected a pair of tandemly duplicated alternative oxidase (AOX) genes, which can serve to oxidise ubiquinol in parallel to complex III. In combination, and taken together with the presence of a standard OXPHOS system, these genes are indicators of flexible aerobic mitochondria, using AOX or complex III to oxidize quinones, or even facultatively anaerobic mitochondria that use fumarate as an electron acceptor [86].

### Kynurenine pathway: present in *Alvinella*, lost in siboglinids

**Fig. 10:**
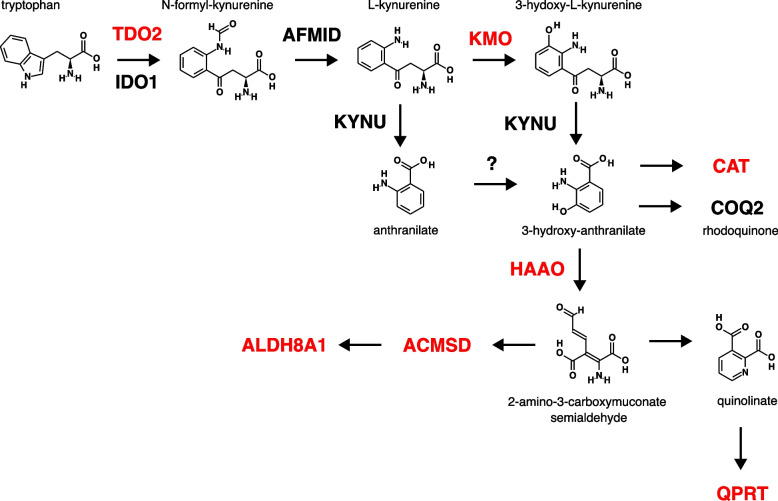
Kynurenine pathway genes. Human gene names are shown in capital letters and those coloured red are present in *Alvinella* and human, but absent from siboglinids. Pathway adapted from KEGG database pathway for tryptophan metabolism [90]

Animal synthesis of rhodoquinone, the electron donor for fumarate discussed in the preceding section, has been shown to be dependent on the kynurenine pathway, via the 3-hydroxyanthranilate (3-HAA) substrate choice by COQ-2 [87–89]. Comparing gene content in *Alvinella* to that of siboglinids, we detected a striking absence in siboglinids (*Riftia, Lamellibrachia* and *Paraescarpia*) of key kynurenine pathway enzymes. We were unable to detect siboglinid orthologs of TDO, KMO, HAAO and QPRT and the associated genes ACMSD, ALDH8A1 and CAT (Fig. 10). All these missing genes are present in *Alvinella pompejana*, although their distribution in Metazoa as a whole is somewhat patchy. Remaining pathway genes, KYNU and IDO, were present in siboglinids, but the overall extent of losses suggests that the pathway is unlikely to function canonically, and its ability, as described, to synthesise 3-HAA is questionable. Siboglinids do encode the ‘e’ exon form of COQ2 (Fig. 9), suggesting that they do use 3-HAA but obtain it via another uncharacterized biosynthetic route, perhaps involving a promiscuous enzyme, or from their symbionts. This may limit their capacity to cope with anaerobic conditions, relative to *Alvinella*.

### A more complete urea cycle

Enzymes involved in urea excretion were reported absent from an early *Alvinella* transcriptome database [14]. In our genome sequence, we found genes for three out of four urea cycle enzymes: argininosuccinate lyase (ASL), argininosuccinate synthetase 1 (ASS1) and arginase (ARG1/2), with the missing exception being ornithine transcarbamylase (OTC). Vestimentiferan siboglinids, but not the bone-eating *Osedax*, encode all four of these urea cycle enzymes [21, 22]. It is notable that *Alvinella* encodes nitric oxide synthase genes, enabling a direct arginine-citrulline connection. Although present in other alvinellids and annelids, we found no NO synthase genes in siboglinids. The significance of this is not readily apparent, but conceivably related to the use of arginine in octopine synthesis (see above).

### Absence of light sensitive proteins

The vents that *Alvinella* inhabits are typically several kilometres below the surface, a depth where there is no ambient light source. We did not detect visual opsins in our predicted gene set, consistent with other deep-sea annelids [22, 91]. In contrast, the deep-sea mussel *Bathymodiolus platifrons* does encode opsin-like genes [92].

We were also unable to detect cryptochrome homologs, light-sensitive proteins that regulate the circadian clock. Cryptochromes appear to be absent from many annelid genomes (including the leech *Helobdella* and siboglinids), although they are present in *Capitella* and *Owenia*. Several other circadian clock components were also undetectable in *Alvinella*, including PER*,* TIMELESS (sensu* tim* in *Drosophila*), CLOCK, and BMAL1 (ARNTL). Although *Alvinella* does not appear to be remarkable in this regard, as the distribution of these genes is patchy within Metazoa as a whole, it is notable that they are present in the deep-sea mussel *Bathymodiolus azoricus *[93]. Against these absences, it should be noted that tidal rhythms are likely to be relevant to deep-sea vent communities e.g. [93, 94].

### An unusual number of carbohydrate sulfo-transferases and esterases

We analysed protein family content using the PANTHER protein database [95], assessing statistically significant enrichment of families in *Alvinella* relative to siboglinids (see the “Methods” section). Many PANTHER families have related or identical functions, so we then used the family classifications to label genes with Gene Ontology (GO) terms from the ‘molecular function’ namespace and calculated the enrichment of GO terms from the genes encoding the significant set of PANTHER families, relative to the complete *Alvinella* protein coding gene set [96] (Table 2).

**Table 2:** Overrepresented PANTHER families in *Alvinella pompejana. Alvinella* annotations were compared to a single combined proteome of the siboglinids *Riftia*, *Paraescarpia* and *Lamellibrachia*. Significant results are shown (α < 0.01, see the “Methods” section). Hits to likely reverse transcriptases, and those where the *Alvinella* sequences were predominantly low complexity have been removed (compare with Additional File 1: Table S8 for raw output)

	***n***	***p*-val**	***p*-adj**	**Description**
**PTHR45953**	35	7E − 23	3.6E − 19	IDURONATE 2-SULFATASE
**PTHR15723**	34	2.6E − 20	8.8E − 17	CARBOHYDRATE SULFOTRANSFERASE 15
**PTHR43806:SF11**	25	1.9E − 18	4.1E − 15	CEREVISIN-RELATED
**PTHR10704**	45	4.2E − 15	7.1E − 12	CARBOHYDRATE SULFOTRANSFERASE
**PTHR47032:SF1**	31	9.7E − 13	1.2E − 09	UDP-D-XYLOSE:L-FUCOSE ALPHA-1,3-D-XYLOSYLTRANSFERASE-RELATED
**PTHR34009**	16	4.6E − 12	5.2E − 09	PROTEIN STAR (Pfam:Methyltransf_21)
**PTHR12137**	39	1.3E − 11	1.3E − 08	CARBOHYDRATE SULFOTRANSFERASE
**PTHR33604:SF3**	24	4.2E − 10	3.9E − 07	OSJNBA0004B13.7 PROTEIN
**PTHR10704:SF44**	12	3.2E − 09	2.7E − 06	PROTEIN, PUTATIVE-RELATED (Pfam:Sulfotransfer_3)
**PTHR46780:SF9**	42	8.6E − 08	6.7E − 05	PROTEIN EVA-1 (Pfam:Gal_Lectin)
**PTHR31511**	12	1.9E − 07	0.00013	PROTEIN CBG23764
**PTHR11022:SF41**	9	4.2E − 07	0.00025	PEPTIDOGLYCAN RECOGNITION PROTEIN 5
**PTHR11214:SF314**	9	4.2E − 07	0.00025	BETA-1,3-GALACTOSYLTRANSFERASE 4
**PTHR32385:SF20**	12	7.9E − 07	0.00045	MANNOSYL PHOSPHORYLINOSITOL CERAMIDE SYNTHASE CSH1-RELATED
**PTHR25462**	25	8.5E − 07	0.00046	BONUS, ISOFORM C-RELATED
**PTHR24028**	35	7.5E − 06	0.0033	CADHERIN-87A
**PTHR47327:SF2**	7	1.1E − 05	0.0047	FI18240P1-RELATED (Pfam:PAN)
**PTHR21461**	10	1.3E − 05	0.0053	UNCHARACTERIZED (Pfam:Glyco_transf_92)

This PANTHER family analysis showed that both carbohydrate sulfotransferases and esterases (Iduronate 2-sulfatase) were over-represented, along with a β−1,3-galactosyltransferase family. This suggests a complex but unexplored sulfate modified carbohydrate chemistry, and it is of note that sulfated glycosaminoglycans are important in tube formation [66]. Sulfotransferases also represent an expanded gene family (27 genes) in vent shrimps together with sulfatases, some of which are positively selected and overexpressed [97]. It seems likely, therefore, that these molecules aid *Alvinella* in its thermal environment or in bacterial symbioses.

Given these hits, enriched GO terms relative to the *Alvinella* protein content as a whole included ‘sulfotransferase activity’, ‘serine-type peptidase activity’ as well as ‘mannosyltransferase activity’ and ‘glycosyltransferase activity’. Notably absent from the results of this analysis were heat shock proteins, for which gene family expansions have been reported in the deep-sea vent mussel *Bathymodiolus platifrons* (HSP70) [92], compared to constitutive high expression in *Paralvinella sulfincola* [98].

## Discussion

A variety of deep-sea vent invertebrates have had their genomes sequenced in recent years, including the mussel *Bathymodiolus platifrons* [92], the clam *Archivesica marissinica* [99], the scaly-footed snail *Chrysomallon squamiferum* [100] and the snail *Gigantopelta aegis* [101]; among annelids, the scale worm *Branchipolynoe longqiensis*, several siboglinids and the alvinellid *Paralvinella palmiformis* [27]; among cnidarians, the deep-sea anemones *Alvinactis idsseensis* sp. Nov. [102] and *Actinernus* sp. [103], but their relative inaccessibility means that they are sparsely represented in the literature, and global studies on adaptations to their unique environmental challenges are not common.

The challenge of living on deep-sea vents begins with the colonization of a patchy and ephemeral habitat, and genome sequences will facilitate future work using population genomics to study community genetic connectivity [104]. Tectonic movements have generated numerous situations of geographical isolation by creating physical barriers to dispersal, particularly through the emergence of microplates or transform faults that have played an important role in the history of species, impacting their dispersal on the seafloor [28, 105, 106]. Allopatric cryptic species are therefore common along oceanic ridges [106–109], but can also be found in other patchy deep-sea habitats [110, 111] and indeed, we find here that *A. pompejana* shows highly divergent populations on either side of the equator. These two lineages showed classic speciation-associated features, with heterogeneous divergence across the genome [112], and asymmetric introgression (e.g. [113, 114] from the North EPR into the South EPR. This introgression was detected in two samples located thousands of kilometres away from the contact zone [29]. Such long-distance introgression may be more frequent along mid-oceanic ridges due to the dynamics of the vent recolonization that often renew source populations following tectonic rearrangements [115].

The best-studied metazoan species in terms of adaptation to high temperatures associated with some vent types is the Pompeii worm *Alvinella pompejana*. Perhaps uniquely among invertebrate Metazoa, *Alvinella* has attracted interest as a possible source of thermostable proteins for structure determination and other analyses. The potential rewards are great if *Alvinella* resources can be used as a tool for the biochemical reconstitution and mechanistic and structural characterization of notoriously challenging mammalian systems, in a manner akin to the role played by fungal genomes of thermophilic species, such as *Chaetomium thermophilum* in eukaryotes [116]. Although *Alvinella’s* protein amino acid composition can be distinguished from other species [8, 15, 16], in a manner that might correlate with ambient temperature, given the paucity of such studies across the full diversity of animals and the corresponding dearth of comparators, it is not clear that definitive evidence has been produced. The analysis presented here instead suggests that *Alvinella* bulk protein amino acid composition is not unusual (Fig. 5), but it is not obvious from protein structural considerations that one should expect a signal at this global level. Neither does it preclude the possibility that some proteins, or groups of proteins sharing particular expression patterns, may be particularly thermotolerant (as suggested by composition of collagen triplets discussed above for instance). Large-scale protein thermal stability assays [117] coupled with more stratified functional groupings and the recent development of computational methods using protein language models to determine folding free energies [118] are likely to help clarify these observations.

By comparing *Alvinella* to other deep-sea vent worms, the siboglinids, we have highlighted similarities and differences in deep-sea survival strategies. We find that both siboglinids and *Alvinella pompejana* encode a rich variety of globins and secreted globins, and that, to an extent, these reflect independent diversifications. We find that *Alvinella*, like the siboglinids and some other but not all deep-sea species, is characterized by the secondary absence of light-sensitive opsins and cryptochromes. Both *Alvinella* and the siboglinids contain enzymes that are characteristic of facultatively anaerobic mitochondria (PNOR and the exon required for COQ2 rhodoquinone synthesis), but that in addition to this, *Alvinella* encodes proteins for octopine synthesis, NO synthesis and a complete kynurenine pathway. These adaptations to anaerobic environments are not unique to deep-sea annelids and are likely inherited from a common annelid or even bilaterian ancestor [85, 119]. Differential gene expression data for *Alvinella* are not available, but the hot vent annelid *Paralvinella sulfincola* shows a reduction in respiratory complex I protein expression at higher temperatures, consistent with a move away from oxidative phosphorylation and increased dependence on anaerobic metabolism, possibly to reduce reactive oxygen species formation [98].

## Conclusions

Evidence suggests that the *Alvinella pompejana* genome has been evolving in a relatively conservative manner. Our analyses have highlighted a typical marine annelid phylogenetic branch length and lack of dramatic genome rearrangement (as opposed to e.g. clitellates) (Figs. 4 and 5), balanced amino acid composition (Fig. 6), an intact Hox cluster and retention of various metabolic genes, lost in other taxa, associated with hypoxic conditions. At the same time, our observations have shown likely adaptations to the deep-sea, such as loss of opsins and cryptochromes and globin gene duplications. Representative genomes of closely related taxa from both deep-sea and less extreme marine environments will help further pin down its specific adaptations.

## Methods

### Sample collection, DNA extraction, sequencing and assembly

*Alvinella* specimens were collected from the top of a chimney at the vent site and the snowball of the Bio9 site at 9°50N/EPR using the telemanipulated arm of the HOV *Nautile* during the French oceanographic cruise Mescal2012 (chief scientist: N. LeBris). Animals were brought back to the surface in an insulated basket and immediately flash-frozen after dissection in liquid nitrogen. Ultra-high molecular weight genomic DNA (gDNA) was extracted following the Bionano Genomics IrysPrep agar-based, animal tissue protocol (Catalogue # 80,002) from the *A. pompejana* gill material of a single animal. Long-read PacBio sequencing and short-read Illumina sequencing were performed at the Genome Centre of the University of California Berkeley using PacBio Sequel II and Illumina Novaseq platforms. An initial assembly was produced using wtdbg2 [120].

### Scaffolding to chromosome level

Additional frozen *A. pompejana* samples corresponding to the anterior part of the worm (head + gills: 10 individuals) were used for further library preparation; sequencing and scaffolding were performed commercially by Dovetail Genomics. Chicago and Dovetail Hi-C libraries were prepared as described previously [121, 122]. The libraries were sequenced on an Illumina HiSeq X to produce 2 × 150 bp paired-end reads. The input de novo assembly, Chicago library reads, and Dovetail Hi-C library reads were used as input data for HiRise, a software pipeline designed specifically for using proximity ligation data to scaffold genome assemblies [121]. An iterative analysis was conducted. First, Chicago library sequences were aligned to the draft input assembly using a modified SNAP read mapper [123]. The separations of Chicago read pairs mapped within draft scaffolds were analyzed by HiRise to produce a likelihood model for genomic distance between read pairs, and the model was used to identify and break putative misjoins, to score prospective joins, and make joins above a threshold. After aligning and scaffolding Chicago data, Dovetail HiC library sequences were aligned and scaffolded following the same method. Illumina sequence reads were used to polish the assembly with ntEdit [124].

### Gene model predictions and genome annotation

A de-novo repeats library was constructed using RepeatModeler v2.0.1 [125] and the assembly was masked for repeats using RepeatMasker v4.1.0 (http://www.repeatmasker.org/). An intermediate masked genome, with low complexity parts left unmasked, was generated for the purpose of gene predictions. Gene models were generated by using a combination of de-novo, homology-based and transcriptome-based approaches. Three bulk RNA-seq samples were mapped to our reference genome using STAR v2.7.1a [126] and transcriptomes were assembled using StringTie v2.1.0 [127]. A de-novo transcriptome was also constructed using Trinity v2.15.0 [128] and clustered using TGICL [129]. This transcriptome was then used in the PASA pipeline [130] to generate a set of transcripts-based gene models. Exonerate v2.4.0 [131] was used to generate a set of protein-based spliced alignments of previously published Alvinellid transcriptomes [16]. These two sets of spliced alignments were used to generate a set of hints to train Augustus v3.2.3 [132] as described in [133]. Augustus generated a set of 17,245 gene models. A final set of 20,098 gene structures was automatically computed as the consensus of all generated evidence using EVidenceModeler v1.1.1 [134] yielding 19,179 protein coding genes.

### Functional annotation of predicted proteins

The set of predicted protein sequences was annotated against publicly available databases. The hmmsearch function from HMMER v3.3 [135] was used to retrieve the Pfam protein domains [136] and the PANTHER families [95]. The presence of a signal peptide in the protein sequences was predicted by using signalP v5.0b [137]. We used diamond v0.9.22 [138] to find the orthologs of our predicted proteins against an instance of NCBI non-redundant protein set downloaded in October 2020 [139]. Finally, the orthologs in the Kyoto Encyclopedia of Genes and Genomes (KEGG) were assigned using the KEGG automatic annotation server blastKOALA [140].

### Mitochondrial genome assembly and annotation

We assembled a complete and circular mitochondrial genome from a set of short Illumina reads using the toolkit GetOrganelle v1.7.7.0 [141]. The toolkit was used as recommended by the authors with background dependencies on Bowtie2 v2.4.1 [142], SPAdes v3.13.1 [143] and Blast + v2.5.0 [144]. The correctness of GetOrganelle’s output was assessed by visualising the assembly graph using Bandage v0.8.1 [145]. Annotation of protein coding, rRNA and tRNA genes was achieved by submitting the assembled genome to the MITOS web server [146].

### Whole genome resequencing

In addition to the reference genome, we included whole genome resequencing (WGS) data from six samples, four from the EPR North (9°50N) and two from the EPR South (18°50S), to explore the genome-wide diversity in *A. pompejana*. Individual DNAs were extracted from tissue samples using the CTAB protocol [147, 148]. WGS libraries were constructed using Illumina Nextera kits. The six libraries were then standardised by DNA shearing to obtain an insert size of 300 to 600 bp using sonication and pooled in equimolar proportion. The pooled libraries were paired-end sequenced (2 × 125 bp) on MiSeq, targeting a coverage of 15 × per sample, at the Genomer platform of Roscoff Biological Station. The data were analysed following the pipeline developed in [149] for a mollusk (*Littorina saxatilis*). In brief, low quality reads (< 15 phred score) and adapter sequences were removed from the raw WGS data using fastp v0.23.1 [150]. Cleaned WGS reads were mapped to the *A. pompejana* reference genome produced in this study using bwa-mem v0.7.17 [151]. Duplicated reads in the alignment file were marked using samtools v1.16 [152]. Mapped reads were processed using mpileup variant caller in bcftools v1.16 [152] to extract single nucleotide polymorphism (SNP) data in variant call format (VCF). Insertions and deletions (Indels), as well as SNPs falling within 5 bp around the indels, were filtered out using bcftools v1.16. Finally, only SNPs with phred quality score of 30, a minimum depth of 5, a maximum depth of 50, and no missing data were included for the downstream analyses using the software vcftools v0.1.16 [153]. This dataset was used to explore the variation across the 6 *A. pompejana* samples using PCA analyses with the R package adegenet [154]. Local PCA analyses were then conducted using a sliding windows approach with windows of 100 kb to explore the variation of population structure along the genome. Finally, phylogenetic analyses were conducted based on the polymorphism data of genomic regions with contrasted PCA patterns with the R package phangorn [155].

### Assessment of putative bacterial contaminations in annelid genomes

We used diamond v0.9.22 [138] to query the predicted protein sequences of selected annelids against NCBI non-redundant protein set downloaded in October 2020 [139]. The predicted proteomes of *Alvinella pompejana* (this study), *Capitella teleta*, *Helobdella robusta* [156], *Lamellibrachia luymesi* [19], *Paraescarpia echinospica *[20] and *Riftia pachyptila* [21] were annotated for putative bacterial contamination. We filtered out every hit to species in the query which were already integrated in the database (here *Capitella teleta* and *Helobdella robusta*) as these species would hamper the proper identification of contaminating protein sequences. We set the significance threshold to *e*-value < 1.e − 20, and the proteins retained as contaminants are those sequences with no significant hit in “Metazoan” and at least 1 significant hit in “Bacteria”. The sequence identifiers of the putative bacterial contaminations are provided in the Supplementary Material, and the statistics are summarised in Supplementary Table S7.

### Amino acid composition

For each genome pair, we calculated reciprocal best hits for predicted proteins using ssearch36 from the FASTA package [157]. For each set of orthologous pairs, we calculated an average amino acid use for each species. Then, for each species, we averaged amino acid usage over all species pair averages. The resulting matrix of average amino acid usage across species was used as input for the ‘prcomp’ PCA function in R and visualised using the ‘fviz_pca_biplot’ function from the ‘factoextra’ library. Similar results along the first PCA were obtained using non-redundant counting of proteins (i.e. no averaging of averages) or using counts taken from conserved columns of protein sequence alignments (not shown).

### Annelid phylogeny

We used OMA [158] to compute the orthologs of predicted proteins of annelids for which the complete genomes were available (listed supplementary table S1) and the outgroup species *Crassostrea gigas*, *Lingula anatina* and *Phoronis australis*. All included datasets (Table S5) were pre-processed to feature only one protein per gene. We selected the 339 single-copy orthologs (118,092 amino acids) which included all annelids and at least one outgroup species to make multiple alignments using mafft-v7.309 [159]. Gap-containing regions were removed using trimAl v1.4.rev15 [160] with the options ‘-gt 1’, and the alignments concatenated. The phylogeny was computed with phylobayes 4.1c using 2 chains with the CAT + GTR model [46, 161].

### Synteny analyses

The orthologous protein sequences *Alvinella pompejana* shared with *Paraescarpia*, *Owenia* and *Mizuhopecten yessoensis* (formerly *Patinopecten yessoensis*) were inferred from pairwise reciprocal best hits using ssearch3 (v3.6) from the FASTA package [157]. Reciprocal best hits were displayed on Oxford grids using the R package macrosyntR [162]. Consistent reciprocal best hits across all species were identified using the method of [163]. The mapping between ancestral linkage groups and genes was obtained from data associated with [164].

### Assembly of DNA likely originating from symbiont

We mapped Illumina paired-end reads from a previous sequencing campaign [26] to our reference assembly using the bwa-mem algorithm of bwa v0.7.17 [151]. Using samtools v1.15.1, we selected the unmapped but paired reads (-f option requiring samflags 13) [165]. We quality-filter and trim the selected reads using cutadapt [166]. These sequences, not contained in the *Alvinella* assembly, were assembled using metaSPAdes [167] and the quality of the produced assembly was evaluated using Quast [168]. Using metabat2 [169], we further binned the contigs into 14 bins of varying quality, assessed using checkM [170].

### Sequence analysis

General sequence database searching was performed with phmmer from the hmmer package [135]. When these produced hits that were clearly distinguishable from other sequence subfamilies, this was taken as evidence of orthology. In other cases, phylogenies were constructed using IQ-TREE [171] following sequence alignment to hidden Markov models (HMM) of the key PFAM representative within the protein of interest [136] and subsequent trimming, as described in [55]*. Globins:* globins were identified by matches to the Pfam Globin HMM. Signal peptides were predicted on these proteins using signalp v5.0 [137]*.* A phylogenetic tree was inferred, cross-referenced with the signal peptide predictions and the monophyletic clade of annelid secreted globins extracted. *Absence of opsins:* predicted sequences were searched using a hidden Markov model (HMM) constructed from opsin-like protein sequences. To be considered opsins, we required that database hits included the conserved Schiff-base Lysine residue. Searches against the Pfam database used gathering thresholds as cutoffs (‘–cut_ga’ in hmmsearch). Presence or absence of octopine dehydrogenases, NO synthases, collagen and tyrosinases was inferred using the Pfam models Octopine_DH, NO_synthase, Collagen and Tyrosinase with gathering thresholds.

### PANTHER family analysis

Proteome sets were searched against the PANTHER 17.0 database, using the supplied pantherScore2.2.pl script with the -B (display best hit), -n (display family and subfamily) and -s (use hmmsearch) options [95]. Enrichment of each PANTHER model was calculated by comparing counts in *Alvinella pompejana* to the summed counts of the siboglinids, using a *Χ*^2^ test. *P*-values were corrected for multiple comparisons using the Benjamini/Hochberg FDR with an alpha of 0.01. *Alvinella* genes with matches to significantly scoring PANTHERs were extracted as a studyset and their associated GO terms were tested for enrichment relative to the GO terms for all *Alvinella* proteins (population) using the ontologizer tool [96].

## Supplementary Information

Additional File 1: Supplementary Tables S1-S9. Table S1: Summary statistics of annelid genome assemblies. Table S2: Summary statistics of the 17 longest Alvinella pompejana scaffolds. Table S3: Summary statistics of Alvinella pompejana genome repeat content. Table S4: Capitella genes reported missing from Lamellibrachia are likely bacterial contaminants. Table S5: Source data of genomes used in annelid phylogeny inference. Table S6: Summary information for metagenomic bins Table S7: KEGG pathway analysis for metagenomic bins. Table S8: The complete table of significant PANTHER hits, including likely reverse transcriptase repeats and low complexity proteins. Table S9: Software used in this study.

Additional File 2: Supplementary Figures S1-S5 & sequences Figure S1: Dovetail Genomics’ link density histogram for the Alvinella pompejana genome. Figure S2: Principal Component Analyses (PCA) performed on the six A. pompejana samples genotyped at 3.33M SNPs. Figure S3: Oxford Grid showing equivalence of Alvinella and Branchiostoma floridae chromosomal segments. Figure S4: Phylogeny of extracellular globins. Figure S5: HIF1A N- and C-terminal Oxygen Dependent Degradation motifs are conserved in Alvinella and siboglinids. Miscellaneous protein sequences for genes mentioned in the text.

## Data Availability

Alvinella genome, protein set, GFF annotation and metagenome protein predictions are available at: doi:10.5281/zenodo.11241339; the raw sequencing reads are associated with the NCBI bioproject PRJNA1291975 [172, 173].

## Abbreviations

3-HAA: 3-Hydroxyanthranilate
ALG: Ancestral linkage group
BUSCO: Benchmarking Universal Single-Copy Orthologs
EPR: East Pacific Rise
FDR: False Discovery Rate
GO: Gene Ontology
HMM: Hidden Markov model
NCBI: National Center Biotechnology Information
NR: Non-redundant
PCA: Principal component analysis
SNP: Single nucleotide polymorphism
VCF: Variant call format
