# Supplementary material for: Chromosome-scale genome assembly and gene annotation of the hydrothermal vent annelid Alvinella pompejana yield insight into animal evolution in extreme environments
Source: BMC Biol. 2025 Sep 2;23:274. doi: 10.1186/s12915-025-02369-7 (PMC12403961; doi:10.1186/s12915-025-02369-7)
Supplement: Supplementary file 2 — Additional File 2: Supplementary Figures S1-S5 & sequences Figure S1: Dovetail Genomics’ link density histogram for the Alvinella pompejana genome. Figure S2: Principal Component Analyses (PCA) performed on the six A. pompejana samples genotyped at 3.33M SNPs. Figure S3: Oxford Grid showing equivalence of Alvinella and Branchiostoma floridae chromosomal segments. Figure S4: Phylogeny of extracellular globins. Figure S5: HIF1A N- and C-terminal Oxygen Dependent Degradation motifs are conserved in Alvinella and siboglinids. Miscellaneous protein sequences for genes mentioned in the text. [file 12915_2025_2369_MOESM2_ESM.pdf]

Supplementary Figures

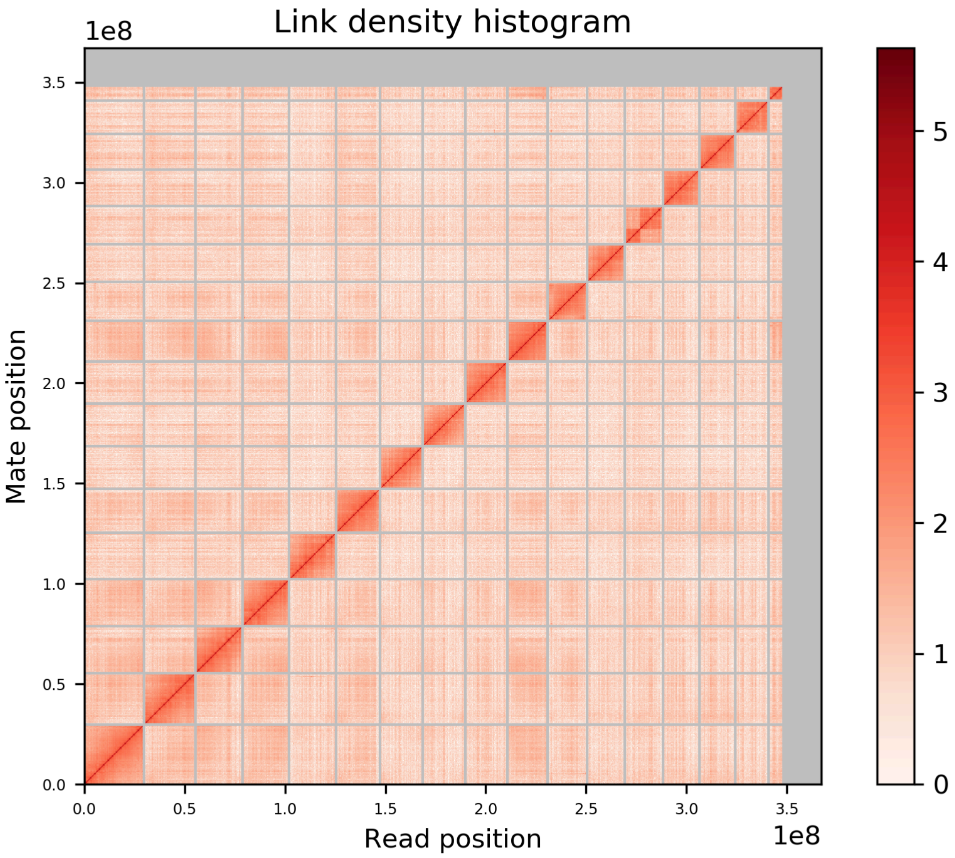

Figure S1: Dovetail Genomics' link density histogram for the *Alvinella pompejana* genome.

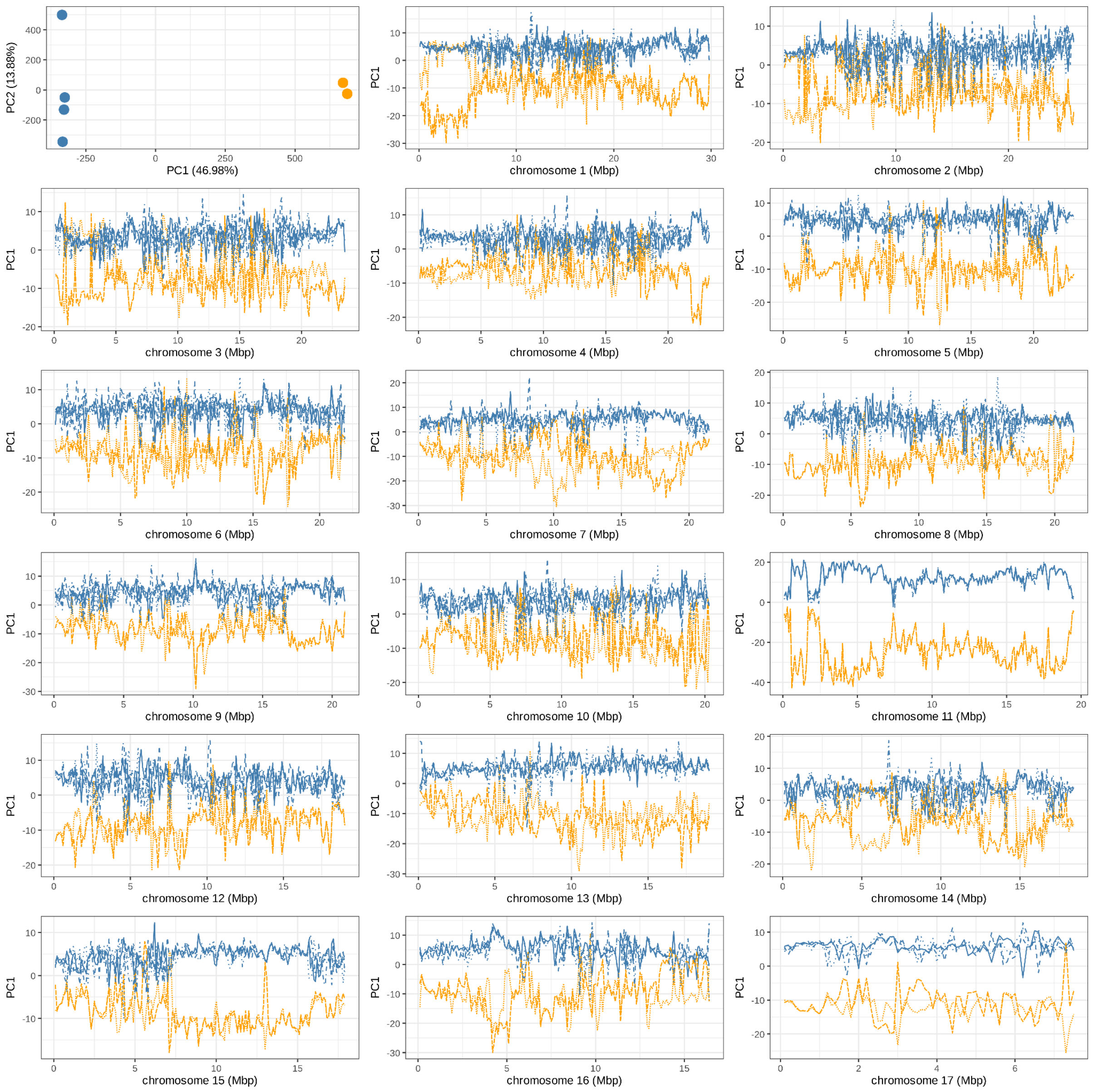

Figure S2: Principal Component Analyses (PCA) performed on the six *A. pompejana* samples genotyped at 3.33M SNPs. The graph on the top left shows the two first axes of PCA computed on the overall dataset. Most of the variation is captured by PC1 (PC1 = 46.98%; PC2 = 13.88%) and distinguishes the two EPR South samples (in orange) from the four EPR north samples (blue). Detailed analyses of genomics regions showing contrasted patterns of divergence between the six samples of *A. pompejana* in a. chromosome 1 and b. chromosome 11. In each subplot, the top graphs show phylogenetic trees computed from the polymorphism data extracted from 1Mbp to 1.5Mbp windows defined from the patterns of divergence observed in the local PCA plot at the bottom. Dotted lines show the limit of the genomics regions that were used to infer the phylogenetic trees. On chromosome 1 (a.), the phylogenies showed, in order of appearance, region of introgression where one EPR south samples is located at mid distance from the EPR north samples and the other EPR south sample, a region of introgression where one EPR south sample clustered perfectly with the EPR north samples, a region of weak divergence where all samples were similarly distant, and a region of high divergence where individuals on either side of the equator were clearly separated. On chromosome 11 (b.), all phylogenetic trees showed clear North – South EPR separation, with the second and last trees also showing very small terminal branches expected from low genetic diversity within each population

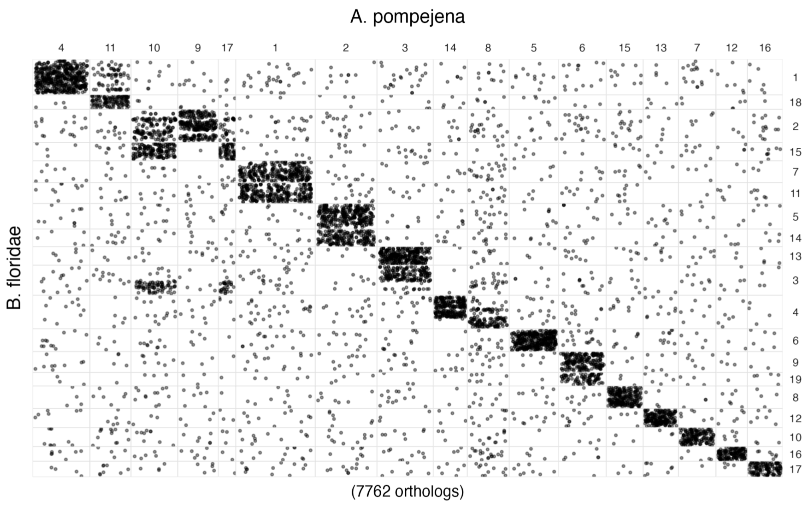

Figure S3: Oxford Grid showing equivalence of *Alvinella* and *Branchiostoma floridae* chromosomal segments.

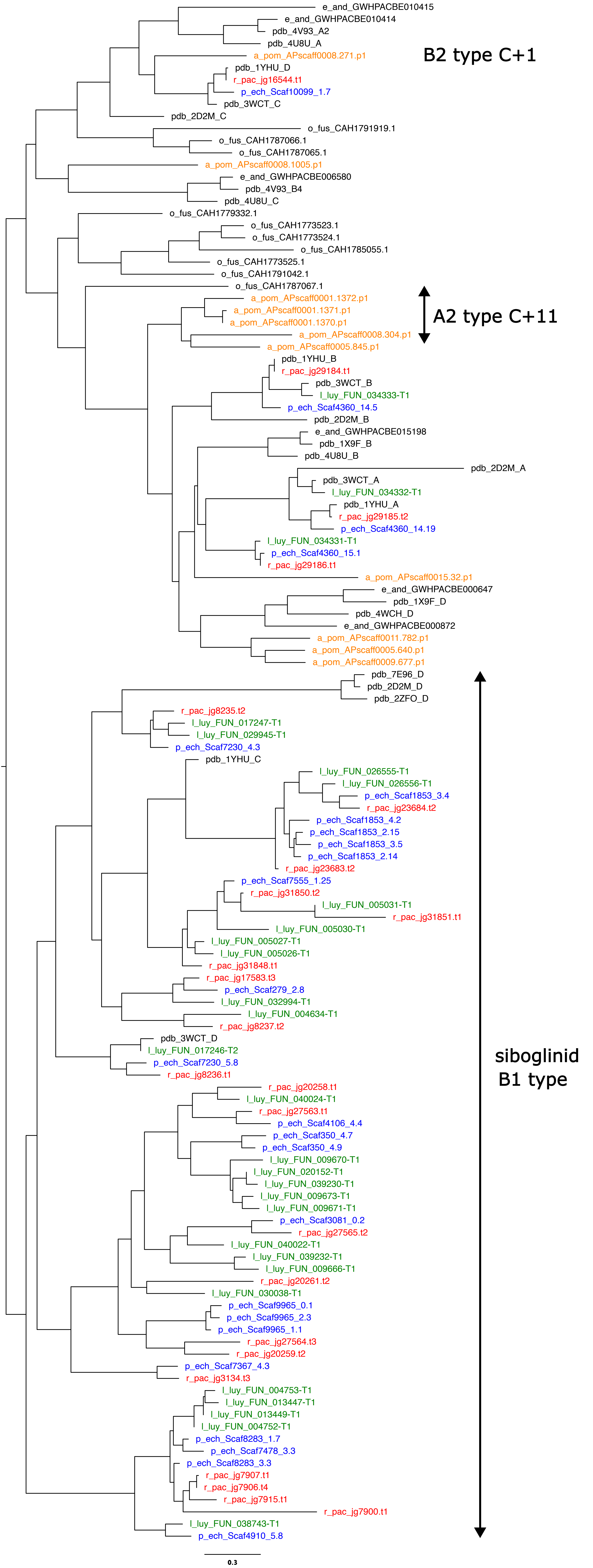
B2 type C+1
A2 type C+11
siboglinid B1 type
0.3
e_and_GWHPACBE010415
e_and_GWHPACBE010414
pdb_4V93_A2
pdb_4U8U_A
a_pom_APscaff0008.271.p1
pdb_1YHU_D
r_pac_jg16544.t1
p_ech_Scaf10099_1.7
pdb_3WCT_C
pdb_2D2M_C
o_fus_CAH1791919.1
o_fus_CAH1787066.1
o_fus_CAH1787065.1
a_pom_APscaff0008.1005.p1
e_and_GWHPACBE006580
pdb_4V93_B4
pdb_4U8U_C
o_fus_CAH1779332.1
o_fus_CAH1773523.1
o_fus_CAH1773524.1
o_fus_CAH1785055.1
o_fus_CAH1773525.1
o_fus_CAH1791042.1
o_fus_CAH1787067.1
a_pom_APscaff0001.1372.p1
a_pom_APscaff0001.1371.p1
a_pom_APscaff0001.1370.p1
a_pom_APscaff0008.304.p1
a_pom_APscaff0005.845.p1
pdb_1YHU_B
r_pac_jg29184.t1
pdb_3WCT_B
l_luy_FUN_034333-T1
p_ech_Scaf4360_14.5
pdb_2D2M_B
e_and_GWHPACBE015198
pdb_1X9F_B
pdb_4U8U_B
pdb_2D2M_A
pdb_3WCT_A
l_luy_FUN_034332-T1
pdb_1YHU_A
r_pac_jg29185.t2
p_ech_Scaf4360_14.19
l_luy_FUN_034331-T1
p_ech_Scaf4360_15.1
r_pac_jg29186.t1
a_pom_APscaff0015.32.p1
e_and_GWHPACBE000647
pdb_1X9F_D
pdb_4WCH_D
e_and_GWHPACBE000872
a_pom_APscaff0011.782.p1
a_pom_APscaff0005.640.p1
a_pom_APscaff0009.677.p1
pdb_7E96_D
pdb_2D2M_D
pdb_2ZFO_D
r_pac_jg8235.t2
l_luy_FUN_017247-T1
l_luy_FUN_029945-T1
p_ech_Scaf7230_4.3
pdb_1YHU_C
l_luy_FUN_026555-T1
l_luy_FUN_026556-T1
p_ech_Scaf1853_3.4
r_pac_jg23684.t2
p_ech_Scaf1853_4.2
p_ech_Scaf1853_2.15
p_ech_Scaf1853_3.5
p_ech_Scaf1853_2.14
r_pac_jg23683.t2
p_ech_Scaf7555_1.25
r_pac_jg31850.t2
l_luy_FUN_005031-T1
r_pac_jg31851.t1
l_luy_FUN_005030-T1
l_luy_FUN_005027-T1
l_luy_FUN_005026-T1
r_pac_jg31848.t1
r_pac_jg17583.t3
p_ech_Scaf279_2.8
l_luy_FUN_032994-T1
l_luy_FUN_004634-T1
r_pac_jg8237.t2
pdb_3WCT_D
l_luy_FUN_017246-T2
p_ech_Scaf7230_5.8
r_pac_jg8236.t1
r_pac_jg20258.t1
l_luy_FUN_040024-T1
r_pac_jg27563.t1
p_ech_Scaf4106_4.4
p_ech_Scaf350_4.7
p_ech_Scaf350_4.9
l_luy_FUN_009670-T1
l_luy_FUN_020152-T1
l_luy_FUN_039230-T1
l_luy_FUN_009673-T1
l_luy_FUN_009671-T1
p_ech_Scaf3081_0.2
r_pac_jg27565.t2
l_luy_FUN_040022-T1
l_luy_FUN_039232-T1
l_luy_FUN_009666-T1
r_pac_jg20261.t2
l_luy_FUN_030038-T1
p_ech_Scaf9965_0.1
p_ech_Scaf9965_2.3
p_ech_Scaf9965_1.1
r_pac_jg27564.t3
r_pac_jg20259.t2
p_ech_Scaf7367_4.3
r_pac_jg3134.t3
l_luy_FUN_004753-T1
l_luy_FUN_013447-T1
l_luy_FUN_013449-T1
l_luy_FUN_004752-T1
p_ech_Scaf8283_1.7
p_ech_Scaf7478_3.3
p_ech_Scaf8283_3.3
r_pac_jg7907.t1
r_pac_jg7906.t4
r_pac_jg7915.t1
r_pac_jg7900.t1
l_luy_FUN_038743-T1
p_ech_Scaf4910_5.8

Figure S4: Phylogeny of extracellular globins. *Alvinella* sequence identifiers are in orange. Siboglinds in red (*Riftia*), blue (*Paraescarpia*) and green (*Lamellibrachia*). Globin subfamilies discussed in the text are labelled.

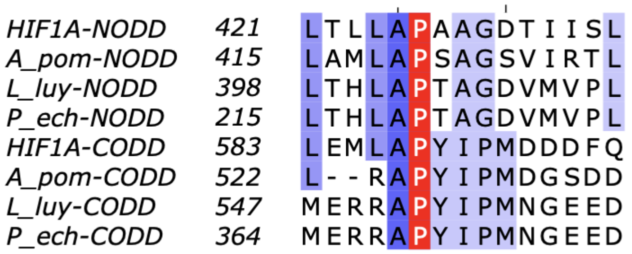

Figure S5: HIF1A N- and C-terminal Oxygen Dependent Degradation motifs are conserved in *Alvinella* and siboglinids. The conserved proline hydroxylation target is shown in red. The human HIF1A sequences are shown with *Alvinella* (A_pom) and the siboglinids (*Lamellibrachia* L_luy, *Paraescarpia* P_ech).

Miscellaneous protein sequences for genes mentioned in text.

Alvinella **Antp** HOX:

```
>a_pom|APscaff0012.403.p1
MSSFYNSPYFDSELNNNFASYSALHYHGLARQNLALQQEFGDPYGNGVDQAAVPHPGTSY
PRFPPYERLDQIRPITSHGGGGGGGGGGGGGGGGGASTHNHMYQQGGHFGLTSPADMGHV
TSAIPNSTKQVASPPPPPPPRAHSSTPARQASPLGRVHGRSDVSPVLPDPIRETGSRDQI
HDHPHPTSVGLYCDNAVKMSVHDNKTDSRATIGTRSPEGSLPAAKDPPANHSPACAEKEK
QSAAGAGAVKEAPSGGESQLPPAESKKSLTDIPDFLSNCKIKDEDIEKMSSMFNDPEMSP
INHRADTPSAAHAQPGTPKSQSAAEDEPGQGQAQTGQGQKNGDSSDEKANSDDGKLRSPE
GAEGDSKSDDNMDEKKDSNSIPMYPWMRSQFGPERKRGRQTYTRYQTLELEKEFHFNRYL
TRRRRIEIAHALCLTERQIKIWFQNRRMKWKKETKQLELLRQAGELPDGLFDDK
```

Alvinella pyruvate:NADP+ oxidoreductase (PNO):

```
>a_pom|APscaff0002.389.p1  GENE.evm.model.APscaff0002.389~~evm.model.APscaff0002.389.p
1  ORF type:complete len:1822 (+),score=442.00 evm.model.APscaff0002.389:1-5466(+)
MWRAWIVGRPVLQTVMRAGCSSRRGLAEITTPRPAMAAVAPAQVKGRPEIRRNENQPWVV
MDGNEAAAYVAYQMSDISFIYPISPATSMGEHMDKWAAQGRKNILGQVVDVNMMQSEAGA
AGALHGAAAAGTLTSTFTASQGLLLMIPNMYLLAGELMPTVFHVSARTVSKHALSIFNDH
SDVMATRQTGFSMLCSASVQEVMDLGVAAHISALKSRLPFLHFFDGYRTSAEMSKIRMMP
PEDIQQIFPYEQVKEHLQKYALNPNSPSIRGTGQRPDIFFQTTVAANRFYNQCPDVVEET
FDEISALTGRKYGLFSYHGSPEADRVAVCMGSASKTLQETVDYLNERGEKTGVVTVHLFR
PWSTKHFLEVLPPSVSKIAVLDRTREDGAVGMPLFLDVNVTMSDAGRNTLITGGQYGLAS
KEFTPAMAKGVFDNLNAPQPKMRYVIGIEDDVTHTHLPYGENIRTTPKSIRQCLFWGLGS
DGTVGANKTAIKTIGLNTDMNAQGHFVYDSHKGDGVTVSHLRFGPEEIKSEYTIQNDADY
LSCSHPSYVYRYEMLEPLKEGGTFVLNSPWTTLEQMEKKLPAHIKNEIAQKKLQFYNIDA
TAIAQSVGLGKRVNMIMQAAFYGLAGVLPQDEAVNLLKKSIETQYSHKGPKVIEMNHKAV
DATMENLTKIQYPESWKDTTEGGSRPGVGNVKKPEFVTNIMDPVLALEGDKLPVSAFVPG
GYQPTGTTKYEKRGIAPAIPVWKPDACTQCNYCSIVCPHAVIRPFLLNKEENKKIPPGFE
ARKAKGGAEVAGYHYTIQVSPYDCTGCEVCVQSCPDDALYMAPFNEVADTYAPHWDYAIS
LPEREEVGDKYTVKGSQFMKPLFEFSGACAGCGETPYLKLATQLFGERMVIANASGCSSV
WGGTSTTIPFSTNREGRGPAWGRSLFEDNAEYGFGMMLATKQRRLKLRQEISAALLEMEL
SDKMKSLFNSYLQYFENPDKCDEVSAAIIAEFEAIGKDNLDPKLRSIYEQRDMLRTQSHW
LVGGDGWAYDIGYGGLDHVFSRGENVNILVLDTEMYSNTGGQVSKATQLSTVAKFATKGK
RQVKKDLGLCAMQYENVYVASVALGANMNQCVQAFKEAEHYNGTSLIIAYAPCIDWGIEM
KNMMKEMKRAVDTGYWSLYRYDPRRAEKGLNPFQLDSKKIKADLEAYLDGQNRFQQLKRS
DKDVAGQLHQHLSADIHHKHDKLMKMSMDNYELLEHLKNLLGETTDTEKVVVLYGSETGN
SAALADVFANELKRRGLRPKCMAMDDFDFDDLPKQDKVFCVVATCGQGEFPGNCKEFWKQ
VSDKELPKDFLKDVQVAVFGMCDRSYVYYNSAAKAFKERFEQLGAKSVMPLGYGDEKDED
RYETAWNEWLPELWNELGTGPPSQELLPPTYSVTMDATGLTVPDVIVPRGSKLLPMMKNV
VLTPPDYDRDIRHYEFDLSGSGFSYSVGDCLGIYPHNNKEDVLKFLDDYGLHSDMVISVQ
DTQGRKDPLPENTTISQLFTEVLDIFGKPARRFYETLSIAAKDEKEKSELEFLLSKDGKD
KLKELTKETVTYADLLRMYPSTKLSLEYLLDHVSRIRPRLYSIASSSEMFGDMLHLCIVK
DDWVTPSGKYRQGLCTRYLQGLSQGSTPDLVAGKMNAAGINIPDSQAPPYVMVALGTGIA
PMRAMIQDREVARMRGESVGPMALFFGARHKRTDYTYGDEFEEWHSGGKGVLNVLSTAFS
RDQAHKIYVQHRIAEHPELIYDYLWKRKGYFYLCGPAGNVPMSVRKAVVDAFVSQGGHSL
AEADKMVTQMQIEGRYNVEAW*
```

Alvinella fumarate reductase (*sensu* yeast OSM1 / FRD):

```
>A_pom|Gene.16723::CL5714Contig1::g.16723::m.16723
Gene.16723::CL5714Contig1::g.16723  ORF type:complete len:516 (+) CL5714Contig1:290-
1837(+)
MSTSSSQTTERVIVVGGGLAGLSAAVEASRHGAKVTIVEKEKQLGGNSAKATSGINGVGT
EAQSAKAIVDDVARFVEDTTKSGAGQSKQELVQVLGRNSAEAHVWLKSFGLNLTDVVQLG
GHSVPRTHRFPPTPDGKPIPVGFTIVSTLRKEVETKLKETVTIVTNAVFKKLLTDGDAVV
GVQYSDESGKLHDVEGTVVLAAGGYANDHTSDSLLVKHVPDLAKLPTTNGPWATGDIIKA
TADLSLSLVNMDRVQVHPTGFIEPKAPNEHTKFLAPEALRGCGAILLDSSGKRFVNELGR
RNYVSDSIFKHGKPYQGNDEYPVVAAMLLTQAVIDKFGPPAIGFYKFKGLIEDVNNLDGV
```

AQKMGVDVAVLKDTIKQYEADAKTGKDQFGKEDFPTVFSENDHFFLAYVTPTLHYCMGGI
EINTDANVLRPGSRIVPGLYAAGEVSGGVHGVNRLGGNSLLECVVFGRIAGRNAAHK*

Pfam **Octopine_DH** containing *Alvinella* sequences:

>a_pom|APscaff0002.759.p1 GENE.evm.model.APscaff0002.759~~evm.model.APscaff0002.759.p1 ORF type:complete len:405 (+),score=35.73 evm.model.APscaff0002.759:1-1215(+)
MITVLVCGGGNGAHVCAGLGASHDNVTTRVLTLYEDEAEKWTKAMGQDGIRITLRHSESD
CLTVVGSPALVTKRAEEAMKPNVDLIIITVPSFAHEQYLKALKPYVKPGMVIVGCPGRAG
FDFAVRSIWAELWSQVSIMNMESLPWACRISKFGCSVDVLGVKETLAGAVQKGEAPTRSA
LDPADMFQKVLGERPRLLTRGHLLGVTLSPTNGCIHPEIMYGRWKDWDGQPMNEPPLFYN
GLDRDTAELISAVSDEVMEIARAIMRQRPQVDLTNVEHIYQWYLRTYPDDIQDKSTLYTS
IRTNKAYKGLVHPCKETEDGRYVPNFKHRYLTEDLPYGMIVLKGIAEVAGVDTPRMDQVI
VWAQRKIGRSFIVGGSLTGEDLDITRSPQRYDFNTLDAITGLTN*
>a_pom|APscaff0002.1541.p1 GENE.evm.model.APscaff0002.1541~~evm.model.APscaff0002.1541.p1 ORF type:complete len:402 (+),score=73.63 evm.model.APscaff0002.1541:1-1206(+)
MVVAVICGGGNGAHVCAGIAASQPGVEARVLTLFADEAERWTNSLKEHDFTVTVHAAKKE
PTKLVAKPTMVTKVPGDAMQGSVDIILFTVPAFAHKQYLEELKPHVKPGMILAGCPGQAG
FEFAVRGIWGDLALQVSVLSFESLPWACRILEFGKSAEVLGTKGTLVGAVSESNPPPKSD
PTATLQKVLGDAPKLVAKGHLLGITLMGTNGYLHPSIMYGKWHKWDGKPFNEVPIFYNGL
DEFSAQVLSDISDEVVATAKAIMEQRPKVDLNNVSHILQWYHRCYGEDIEDKSTLYTCIR
TNRAYKGLTHPCVKNDDGTYVPNFKYRYLTEDIPFGLVVMRGIASIAGVQTPNMDKVITW
AQKQLGKEYLVDGQLKGNDLNETRCPQRYGLESLDKVLGLA*

Alvinella **urea cycle** proteins:

argininosuccinate lyase:

>a_pom|APscaff0004.737.p1 (**ASL1**)
GENE.evm.model.APscaff0004.737~~evm.model.APscaff0004.737.p1 ORF type:complete
len:578 (+),score=116.81 evm.model.APscaff0004.737:1-1734(+)
MRRRRLNMSMWKECSTLSATTSEKSATSNNRRKRRNFFDEIQQLMPSCVKRLHFNNEQEV
PRYAECLDLSEESIDQSDEDGDAGDSGGGGGGEQLEECFSCKVLDAINKASTKMAETNKG
GKLWGGRFTGTTDPIMHEFNASISYDKCMWKADIQGSKAWVSALLKAGLVTEEEKELITS
GLSKISDEWAAGTFSLEPTDEDIHTANERRLKELIGPVGGKLHTGRSRNDQVSTDMRLWL
RESIGNMKNLLKTLIAVFVSRAKREISILMPGYTHLQRAQPIRWSHWLLSYASMLQRDYE
RLDSLTPRVNTLTLGSGALAGNPFNIDMNLLAEKLGMERISLNSLDAASDRDFIAEFLFW
ASLTSVHLSRWAEDLILYSTAEFGFVTMSDAYSTGSSMMPQKKNADSLELIRGKAGHVYG
QCTILMVTMKGLPSTYNKDLQEDKQAMFDVYDTLTGVMQVAAGVLSTLKINADKCQRALS
LDMLATDIAYYLVRKGMPFREAHSLSGKCVALAEKRGCTLDKLSLKEFNDIHPLFTEDVM
KLWDFESSVEQYQSPNGTSSSSVLSQINILETWLNSK*

argininosuccinate synthetase 1:

>a_pom|APscaff0003.931.p1
(**ASS1**) GENE.evm.model.APscaff0003.931~~evm.model.APscaff0003.931.p1 ORF
type:complete len:410 (+),score=67.30 evm.model.APscaff0003.931:1-1230(+)
MSTKDTVVLAYSGGLDTSCILKWLQEKGYDVITFTANVGQDEDFDAARAKAEKLGAKKVV
IQDLRQEFFEEFISVGIQANAIYEDRYLMGTAFARPCIARAIVKVAKEEGAKYISHGATG
KGNDQVRFELACYALYPEVKLISPWRLPEFYTRFRGRPDLFKYAEEHGIPLPVSPKAPWS
IDANMMHVSYESGILEDPRNEAPATLYEMTTDPTKAPDSPERLVIEFKNGIPVKVKNLND
NTEISGGLNLYMYLNKIGSRHGVGRIDIVENRFLGMKSRGIYETPAGTILYQAHLDIENL
TMDRELRKIKQQLSVQFSEQVYRGFWFSPECAFVRHCIAKSQEGVDGTVYVKVYKGNVYI
TSRESACSLYNQELVSMDVQGNYDPSDAAGFIIVNALRLKEYNRRQIQK*

arginase:

>a_pom|APscaff0004.221.p1 (**ARG1/2**)
GENE.evm.model.APscaff0004.221~~evm.model.APscaff0004.221.p1 ORF type:complete
len:322 (+),score=25.06 evm.model.APscaff0004.221:1-966(+)

MSVFDKPVGVVGIPFDKGQPRKGVNHGPEVLRRAGAVSIIEELGYDVTDYGDIRLEDVPN
DPPAFGVKLPRTIGGDMKRLSDKVSQVVASGAICLNLGGDHTLGIGSINGHLAAKPHAAV
IWIDAHADLNPPTSSPSGNIHGMPLAFLIKEVQRYMPQLPGFEWLKARLNAQDVAYIGIR
DVDKAEKKLIKELGITYYDMYDIDRMGIHQVVEGALNAVNPRNDRPIHLSFDIDALDACY
CPSTGTPVSAGLTLREGMYIVEKIFRTGNLSVFDIAEVNPKLGSPEDVAVTVKSALSLIG
AAFGQRTVDFLADDYTIPKPN*

Cluster of intracellular globins:

```
>a_pom|APscaff0005.650.p1
MGCAPSKEGATFTSSDPQPANSSQSRSSGASKSILREAVESGSYSKMASYKPDPRCPLTE
RQLYSITKSWKAINREMASTAVNMFVRLLEFDGIRSFFSKFKDTQTVAELRANKVFEGHA
LSVISIIDEVITNLDDMDYVISLLQATGESHSIKFENFNPDFLWKVEGAFLWAVKETLGD
RYTISIENIYTTTIRYIIQSLHDAFTKHRKENPRESTETEKAGELPTNETETHGETKAIS
DNSLSVDFIGTNGATKHFVAAEE*
>a_pom|APscaff0005.649.p1
MGCAPSKEGATVKARDVHPATNSLSRSTDASKSILREAVETGSYSKMASYKPDPRCPLTE
RQLYSITKSWKAINREMASTALNMFVRLLEVRGIRAVFSKFKDHTTVAELRADAIFQSHA
LSVISVIDEVITNLDDMDYVISLLQATGESHSIQTENFQADYLWNVEGAFLWAVKETLGD
RYTISIEQIYVVTIRYILKSLHASFTEHRANRARAPTGGKNTQ*
>a_pom|APscaff0005.651.p1
MGCAPSKEGATVKAVDLQAVNSSQSRSSGASKSILREAVETGSYSKMASYKPDPRCPLTE
RQLYSITKSWKAINREMASTAVNMFIRLLEHDGIRSFFTKFKDHKTVAELRASKVFESHA
LMVISVIDDVITNLDDMDYVMSLLQATGESHSIKFKNFNPDFLWNVEGAFLWAVKETLGD
RYTISIENIYTITIRYILQSLHDAFTKHRERQNSTNNDSKKTNLLNQELSTADRKTAPDS
KD*
>a_pom|APscaff0005.652.p1
MGCAPSKEGATVTSGDTQRANGSQSRSSCATKSILREAVESGSYSKMASYKPDPRCPLTE
RQLYSITKSWKAINREMASTAVNMFVRLLEVDGMRSFFSKFKDHKTVAELRASKVFETHA
LMVISVIDDVITNLDDMDYVISLLEATGESHSKKFGNLNADFLWNVEEPFLWAIRETLGD
RYTISIENIYTITIRYILQALHDSFTKHRQGQTTAETETRSNEKQAEVEDTIGEKDKDLP
YDRMSSTEIKGKYYDEEIKPKLSVTSDKPKETVRN*
>a_pom|APscaff0005.653.p1
MGCAPSIEGATVTSGDVQPAKSSLSKSTGATKSILREAVDSGRYSRMASYKPDPRCPLTE
RQLYSITKSWKAINREMASTAVNMFVRLLEIDGIRSFFSKFKDHKTVAELRASEVFEGHA
LMVISAIDDVITNLDDMDYVISLLEETGQSHSRRFHSFNPAFFWKVEGAFLWAVKETLGD
RYTISIENIYTTTIRYILQSLHDAFVGHRGNHADDDPAKSESLLQHQEKKDEEEAAGPEK
PRTNEE*
```

Protein with S/G/A amino acid composition percentages most similar to tube:

```
>a_pom|APscaff0012.608.p1
MSVSAGCGKNHFSKSTSASGSYAIAQGKNGVCTVTASFSQSTSASAGCGKNHWSKSGSAS
GSYAMKKGKGGKLICKTGSYSCSTSVSAGVGKNHFSKSTSASGSYAVTQGKNGVCTCIAS
FSRSVSTSAGCGKNHYSMSASASGSVAKKTGKGGKLICQTGRFSSSKSVSAGVGKNHFSK
STSASGSYAIQKGKNGVCTVVSSFSRSVSTSVGCGKNHYSMSGSASGSFAKKTGKGGKLI
CQTGRYSSSTSVSAGVGKNHYSKSASLSTSYAIQKGKNGVCTVTSSFSRSVSTSVGCGKN
HFSMSGSASGSFAKKTGKGGKLICKTGRYSSSKSVSAGVGKNHYSKSTSVSGSYLVAQGK
GGACTVISSFSKSVSASAGCGKNHWSKSGSCSGSYALKRGKGGKLVSETGRFSNSMSVSA
GCGKNHFSKSTSASGSYAIAQGKNGVCTVTASFSQSTSASAGCGKNHWSKSASASGSYGV
KRGKGGKLLSATGRYSSSMSASAGCGKNHWSNSASASGSYAVSLGAGQPCDA*
```
